# Insights on the regulation and function of the CRISPR/Cas transposition system located in the pathogenicity island VpaI-7 from *Vibrio parahaemolyticus* RIMD2210633

**DOI:** 10.1128/iai.00169-25

**Published:** 2025-05-01

**Authors:** Jesús E. Alejandre-Sixtos, Kebia Aguirre-Martínez, Jessica Cruz-López, Aliandi Mares-Rivera, Samanda M. Álvarez-Martínez, David Zamorano-Sánchez

**Affiliations:** 1 Programa de Microbiología Genómica, Centro de Ciencias Genómicas, Universidad Nacional Autónoma de México, Cuernavaca, Morelos, México; University of California Davis, Davis, California, USA

**Keywords:** CRISPR/Cas, pathogenicity islands, transcriptional regulation, *Vibrio parahaemolyticus*, transposons

## Abstract

CRISPR/Cas-mediated transposition is a recently recognized strategy for horizontal gene transfer in a variety of bacterial species. However, our understanding of the factors that control their function in their natural hosts is still limited. In this work, we report our initial genetic characterization of the elements associated with the CRISPR/Cas-transposition machinery (CASTm) from *Vibrio parahaemolyticus* (*Vpa*CASTm), which are encoded within the pathogenicity island VpaI-7. Our results revealed that the components of the *Vpa*CASTm and their associated CRISPR arrays (*Vpa*CAST system) are transcriptionally active in their native genetic context. Furthermore, we were able to detect the presence of polycistrons and several internal promoters within the loci that compose the *Vpa*CAST system. Our results also suggest that the activity of the promoter of the atypical CRISPR array is not repressed by the baseline activity of its known regulator VPA1391 in *V. parahaemolyticus*. In addition, we found that the activity of the promoter of *tniQ* was modulated by a regulatory cascade involving ToxR, LeuO, and H-NS. Since it was previously reported that the activity of the *Vpa*CAST system was less efficient than that of the *Vch*CAST system at promoting transposition of a miniaturized CRISPR-associated transposon (mini-CAST) in *Escherichia coli*, we analyzed if the transposition efficiency mediated by the *Vpa*CAST system could be enhanced inside its natural host *V. parahaemolyticus*. We provide evidence that this might be the case, suggesting that there could be host induction factors in *V. parahaemolyticus* that could enable more efficient transposition of CASTs.

**IMPORTANCE:**

Mobile genetic elements such as transposons play important roles in the evolutionary trajectories of bacterial genomes. The success of transposon dissemination depends on their ability to carry selectable markers that improve the fitness of the host cell or loci with addictive traits such as the toxin-antitoxin systems. Here we aimed to characterize a transposon from *Vibrio parahaemolyticus* that carries and could disseminate multiple virulence factors. This transposon belongs to a recently discovered family of transposons whose transposition is guided by crRNA. We showed that the transposition machinery of this transposon is transcribed in *V. parahaemolyticus* and that there are likely host-associated factors that favor transposition in the natural host *V. parahaemolyticus* over transposition in *Escherichia coli*.

## INTRODUCTION

**Fig 1:**
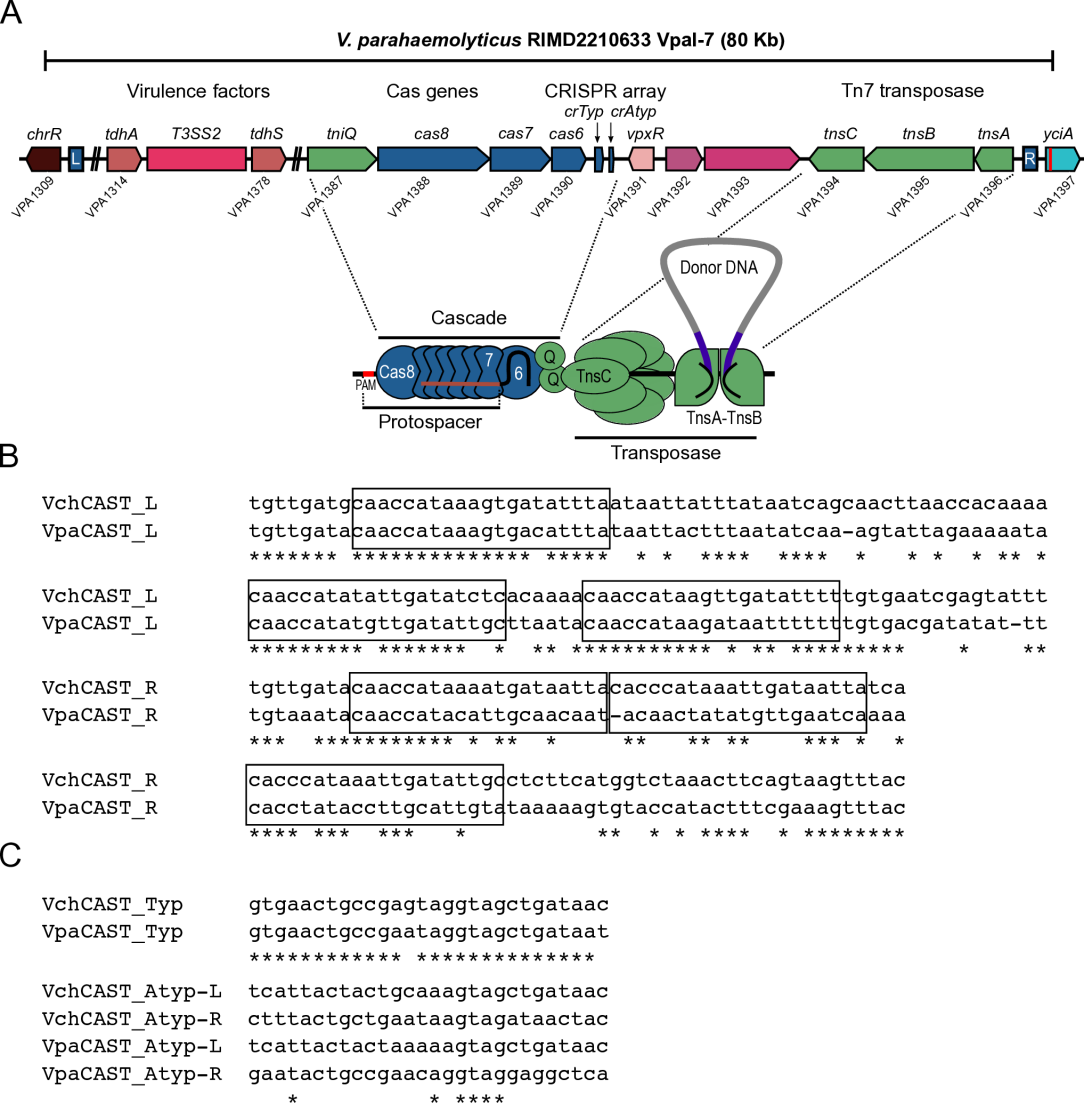
The pathogenicity island VpaI-7 from *V. parahaemolyticus* RIMD2210633 is within a CRISPR/Cas-transposon. (**A**) Diagram depicting genes encoding relevant components of the VpaI-7 island including virulence factors and the elements of a Type IF3 CRISPR/Cas transposition system. (**B**) Sequence alignment of the predicted TnsB-binding sites (TnsB-box) in the L- and R-terminal regions of the *Vch*CAST and *Vpa*CAST systems. The TnsB-boxes are delimited by rectangles. (**C**) Sequence alignment of the direct repeats flanking the spacer sequences of the typical and atypical CRISPR arrays from the *Vch*CAST and *Vpa*CAST systems.

*Vibrio parahaemolyticus* is a cosmopolitan bacterium capable of colonizing the intestines of marine animals such as shrimp and causing gastroenteritis in humans (1). Its virulent properties are mainly associated with the production of three hemolysins (TDH, TRH, and TLH) and two type 3 secretion systems, one located on chromosome 1 (T3SS1) and another on chromosome 2 (T3SS2) (2, 3). Two copies of the thermostable direct hemolysin and the T3SS2 are encoded in the pathogenicity island VpaI-7 (2) (Fig. 1A). It has been proposed that this island was acquired by horizontal gene transfer (HGT) recently in the evolutionary history of the pandemic clone of *V. parahaemolyticus* O3:K6 during a pandemic outbreak that occurred between the years 1995 and 1996 (4–6). The continued dissemination of virulence factors through the horizontal transfer of VpaI-7 could result in the emergence of bacterial strains with greater pathogenic capacity (7, 8). To date, the molecular mechanisms that allowed the spread of the VpaI-7 island in *V. parahaemolyticus* strains remain unknown, as well as whether it retains the ability to move by horizontal transfer.

Recently, a series of transposons of the Tn7 family whose transposition mechanism involves the joint activity of a TnsABC transposase, a TniQ family protein, and a minimalistic CRISPR/Cas system has been characterized (9). These types of transposons are commonly referred to as “CRISPR-associated transposons” (CASTs) (10–12) and are widely distributed among a vast variety of bacterial genera including the *Vibrio* genus (13, 14). Minimalistic CRISPR/Cas systems, unlike canonical CRISPR/Cas systems, lack the proteins necessary for CRISPR array adaptation and target interference, processes involved in immune memory mechanisms and defense against foreign DNA, respectively (13). The minimalist CRISPR/Cas systems encoded within CASTs have the function of guiding the transposition of these mobile genetic elements through the base complementarity of a CRISPR RNA (crRNA) with their double strand DNA target site known as the protospacer sequence (15) (Fig. 1A). The transposition of CASTs encoding minimalistic CRISPR/Cas I-F systems depends on the CRISPR-associated complex for antiviral defense (Cascade) composed of the Cas8, Cas7, and Cas6 proteins (16) (Fig. 1A). The Cascade I-F recognizes a 5′-CC protospacer-adjacent motif (PAM), and the crRNA forms a DNA-RNA double strand through base complementarity with the protospacer to promote the transposon insertion event (15) (Fig. 1A). Once the Cascade complex recognizes its target, it uses TniQ as an intermediary to recruit the transposase composed of TnsA, TnsB, and TnsC (16) (Fig. 1A). The TnsA-TnsB complex recognizes the asymmetric right (R) and left (L)-terminal sequences, which have multiple TnsB-binding sites and delimit the CAST, while TnsC acts as a regulator of transposition by acting as a bridge between TniQ and TnsA-TnsB (17–21) (Fig. 1).

Sequence analysis of the VpaI-7 island strongly suggests that it is a CAST, and it is flanked by Tn7-like R- and L-terminal sequences and encodes the proteins TnsA (VPA1396), TnsB (VPA1395), TnsC (VPA1394), TniQ (VPA1387), and a minimalistic CRISPR/Cas system I-F3a composed of the proteins Cas8 (VPA1388), Cas7 (VPA1389), and Cas6 (VPA1390), as well as two independent CRISPR arrays, named “typical” and “atypical” (22) (Fig. 1). The R- and L-terminal sequences have putative TnsB-boxes that share sequence identity with the TnsB-boxes previously characterized for the *Vch*CAST system encoded in the Tn6677 transposon from *V. cholerae* HE-45 and homologous Type I-F3 CAST systems (23). The typical CRISPR array of VpaI-7 is composed of three direct repeat sequences and two spacer sequences complementary to plasmid sequences found in various species of the *Vibrio* genus (24, 25).

The atypical CRISPR array contains two imperfectly repeated sequences flanking a single spacer (22, 23). The spacer of the atypical array has sequence complementarity with the *yciA* gene (VPA1397) (22, 23), which is the gene located downstream of the R-terminal sequence of the CAST on chromosome 2 from *V. parahaemolyticus* (Fig. 1A). It has been documented that CASTs are typically transposed in an R-L orientation (16, 26). This suggests that the spacer of the atypical array allowed the insertion of the CAST harboring the VpaI-7 island.

The CAST transposition mechanism has been mainly studied in the heterologous background of *Escherichia coli* (16, 22, 23, 26), outside of its native context. The first CRISPR-mediated transposition system studied experimentally was the one encoded in the Tn6677 transposon from *V. cholerae* HE-45 (16). The CRISPR/Cas-transposition machinery (CASTm) of this CAST was found to be functional in *E. coli* and was used to generate a genetic tool, named INTEGRATE, for the generation of programmed mini-CAST insertional mutations (26). The INTEGRATE tool is practical for studying CAST transposition since it consists of a single plasmid containing a programmable CRISPR array, the *tniQ*, *cas8*, *cas7*, *cas6*, *tnsA*, *tnsB,* and *tnsC* genes, all under the control of the strong synthetic promoter P_J23119_ (26). An interchangeable mini-CAST genetic cargo flanked by the R and L-terminal sequences is present on the same plasmid (26). Using INTEGRATE-inspired approaches, previous studies reported that the CRISPR-associated transposition system encoded in VpaI-7 (*Vpa*CAST system) is functional in *E. coli*, although with lower efficiency than the system encoded in Tn6677 (22, 23, 26). Little is known about the function and regulation of CAST systems such as the *Vpa*CAST system in their native genetic background. In this regard, it was recently reported that the product of the gene VPA1391, located downstream of the CRISPR arrays within VpaI-7, is a conserved Xre-type transcriptional regulator that exerts a negative effect on the expression of the atypical array (22). The evidence presented was obtained through biochemical experiments and genetic studies in the heterologous host *E. coli* (22); therefore, the exact contribution of this regulator in controlling the expression of the atypical array crRNA (crAtyp) in its native genetic background is unknown so far. Furthermore, to our knowledge, there are no studies directly evaluating the transcriptional activity, regulation, and arrangement of the CAST system elements of the VpaI-7 island.

In this work, we asked some fundamental questions regarding the physiological relevance of the *Vpa*CAST system in *V. parahaemolyticus*. We asked (i) whether the elements of the *Vpa*CAST system are transcribed under standard laboratory growth conditions; (ii) whether the active promoters of the components of the *Vpa*CAST system are subject to transcriptional regulation; and (iii) whether the components of the *Vpa*CASTm are equally efficient to promote transposition in its native genetic background as in the heterologous host *E. coli*. The observations and discoveries reported in this work will open new areas of inquiry that will lead to a better understanding of the contribution of CRISPR/Cas-mediated transposition to the evolution of *Vibrio* spp. pathogenicity and colonization success.

## RESULTS

### The genes involved in CRISPR/Cas-mediated transposition are transcriptionally active in *V. parahaemolyticus* RIMD2210633

**Fig 2:**
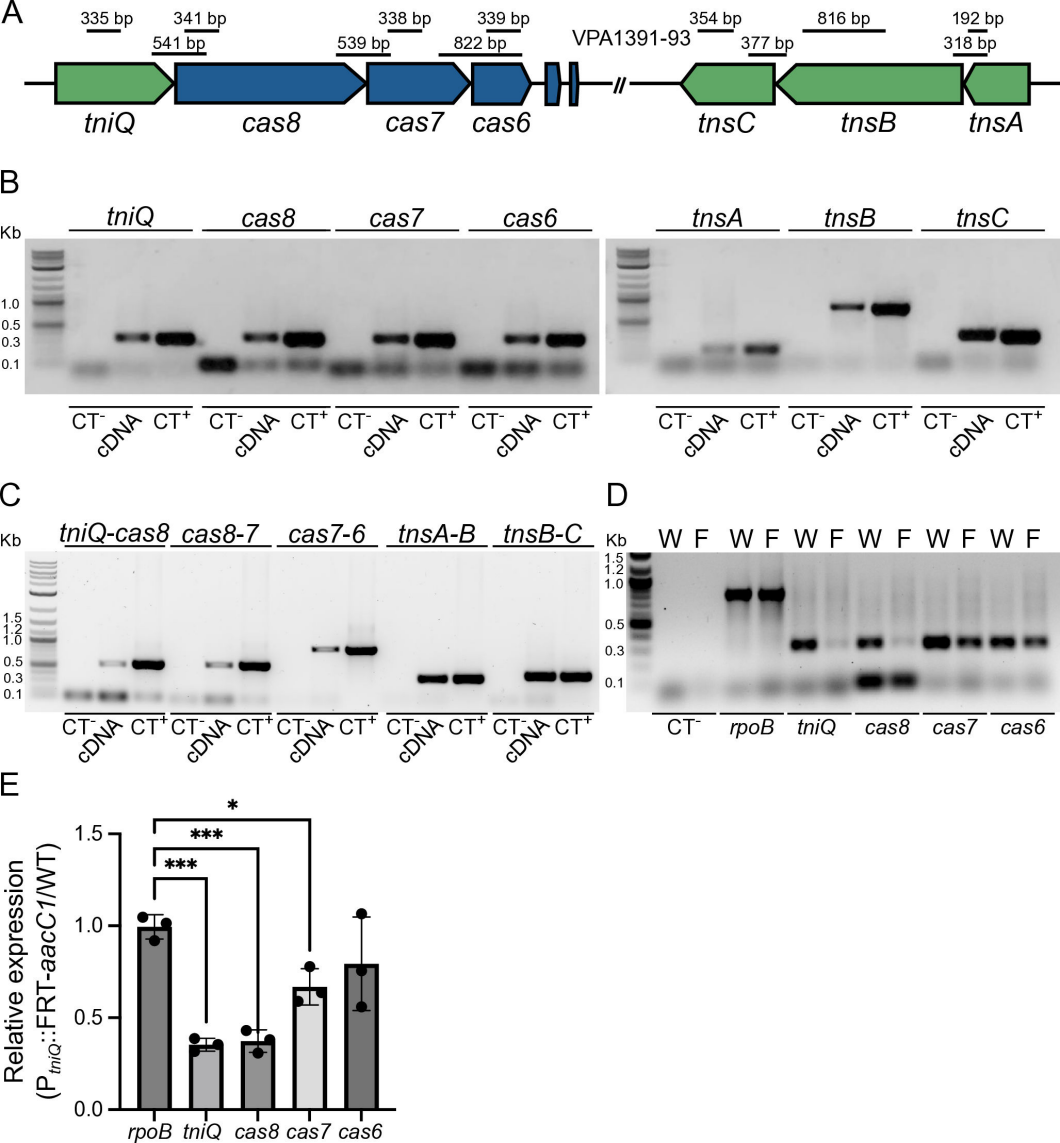
The genetic clusters *tniQ-cas876* and *tnsABC* are transcribed as polycistrons in *V. parahaemolyticus* RIMD2210633. (**A**) Diagram illustrating the arrangement of the *tniQcas876* and *tnsABC* genetic clusters. The Tn7-like transposition-associated genes are colored in green, and the CRISPR/Cas-associated genes are colored in blue. The lines shown above the genetic clusters indicate the location of the regions amplified by PCR, and the size of the amplification product is also indicated. (**B, C**) Image of the electrophoretic migration in an agarose gel of PCR products obtained with primer pairs specific to (B) monocistrons or (C) polycistrons. (**D**) Image of a representative RT-PCR analysis of the transcript abundance of *rpoB*, *tniQ*, *cas8*, *cas7,* and *cas6* in the WT strain (W) or the P*_tniQ_*::FRT-*aacC1* mutant strain (**F**). Total RNA was used as a template in negative controls (CT^-^). For panel (D), a primer pair used to amplify a region of *rpoB* was used to assess the contamination of total RNA with gDNA. Complementary DNA (cDNA) obtained by RT-PCR was used as a template to detect the transcripts of interest. Genomic DNA from *V*. *parahaemolyticus* RIMD2210633 was used as a template in positive controls (CT^+^). (**E**) Bar graph representing the mean and standard deviation of the relative abundance (P*_tniQ_*::FRT*aacC1*/WT) of transcripts for *rpoB*, *tniQ*, *cas8*, *cas7,* and *cas6* obtained through a semiquantitative RT-PCR analysis. Means were compared using an ordinary one-way ANOVA test followed by a Dunnett’s test to compare each mean to the mean of the control *rpoB*. Mean differences with a *P* value ≤ 0.05 were interpreted as significant. * Adjusted *P* value ≤ 0.05, *** ≤0.001.

To begin to address the physiological relevance of the *Vpa*CAST system in *V. parahaemolyticus* RIMD2210633, we first evaluated whether its components were transcriptionally active under standard laboratory growth conditions. The apparent organization of the genetic context of these genes suggests that *tniQ*, *cas8*, *cas7,* and *cas6* are part of a four-gene operon encoded on the leading strand, while *tnsA*, *tnsB,* and *tnsC* are forming an operon encoded on the lagging strand (Fig. 2A). An RT-PCR analysis revealed the presence of transcripts corresponding to *tniQ*, *cas8*, *cas7*, *cas6*, *tnsA*, *tnsB,* and *tnsC* (Fig. 2B). We also found potential polycistronic transcripts containing *tniQ-cas8*, *cas8-cas7*, *cas7-cas6*, *tnsA-tnsB*, and *tnsB-tnsC* (Fig. 2C). The amplification of the putative *tniQ-cas8*, *cas8-cas7,* and *cas7-6* polycistronic units was weaker compared to the amplification of the polycistronic units corresponding to *tnsA-tnsB* and *tnsB-tnsC*.

To further analyze the *tniQcas876* operon, we compared the expression of these genes in a WT strain and a strain that has an insertion of an FRT-*aacC1* cassette immediately upstream of the first codon of *tniQ* (P*_tniQ_*::FRT-*aacC*1), most likely displacing its promoter. The *aacC1* gene, which encodes a gentamicin 3-N-acetyltransferase, is positioned in a divergent orientation with respect to *tniQ*. We isolated total RNA from the WT strain and the P*_tniQ_*::FRT-*aacC*1 mutant strain in three independent experiments and did a semiquantitative RT-PCR analysis to evaluate the transcript abundance of *rpoB*, *tniQ, cas8, cas7,* and *cas6*. RpoB is the β subunit of the RNA polymerase, and its expression should be the same in the WT strain and the P*_tniQ_*::FRT-*aacC*1 mutant strain. As expected, our results showed that the expression of *rpoB* was not altered when comparing the WT strain and the P*_tniQ_*::FRT-*aacC*1 mutant strain (Fig. 2D and E). By contrast, the expression of *tniQ* and *cas8* decreased in the P*_tniQ_*::FRT-*aacC*1 mutant strain when compared to the expression in the WT strain (Fig. 2D). We observed a modest decrease in the abundance of *cas7* transcripts in the P*_tniQ_*::FRT-*aacC*1 mutant strain compared to the WT strain but could not detect significant changes in the expression of *cas6* with the resolution of our assay (Fig. 2D and E). Regardless, our results suggest that the transcriptional activity of P*_tniQ_* mainly affects the expression of *tniQ* and *cas8* and that the *tniQcas876* genetic cluster could contain complex operons with internal promoters.

We next used the bacterial sigma70 promoter recognition program BPROM (27) to predict putative promoters upstream of *tniQ*, *cas8*, *cas7*, *cas6*, *tnsA*, *tnsB,* and *tnsC*, and the results are shown in Table 1. The BPROM tool predicted promoters upstream of all the genes analyzed, with different degrees of conservation with respect to the consensus of the –10, –10 extended, and −35 boxes of sigma70 promoters from well-characterized *E. coli* genes. The canonical A and T nucleotides located at positions −11 and −7 of the −10 box, respectively, are conserved in all predicted promoters. These nucleotides are important for the formation of the open complex and transcription initiation at the −10 box of sigma70 promoters (28).

**TABLE 1:** Predicted promoters within the putative *tniQcas876* and *tnsABC* operons

Gene name	−35 box (consensus: TTGACA)	−10 extended and −10 box (consensus: TGNTATAAT)	Centered position of the promoter with respect to the start codon
*tniQ*	TTGGTG	TAATAAAAT	−72
*cas8*	TTGTCT	TTTCAATCT	−53
*cas7*	ATGCTT	CGGTATCAT	−117
*cas6*	TTGTTA	AGCCACGAT	−266
*cas6*	TTAATG	TTGCAAACT	−56
*tnsA*	TTGTAA	TGCAACAAT	−63
*tnsB*	TTGATT	AGTCATGAT	−35
*tnsC*	TTAAAG	CGATATCAT	−235
*tnsC*	ATGTTG	CTCTAAACT	−155

**Fig 3:**
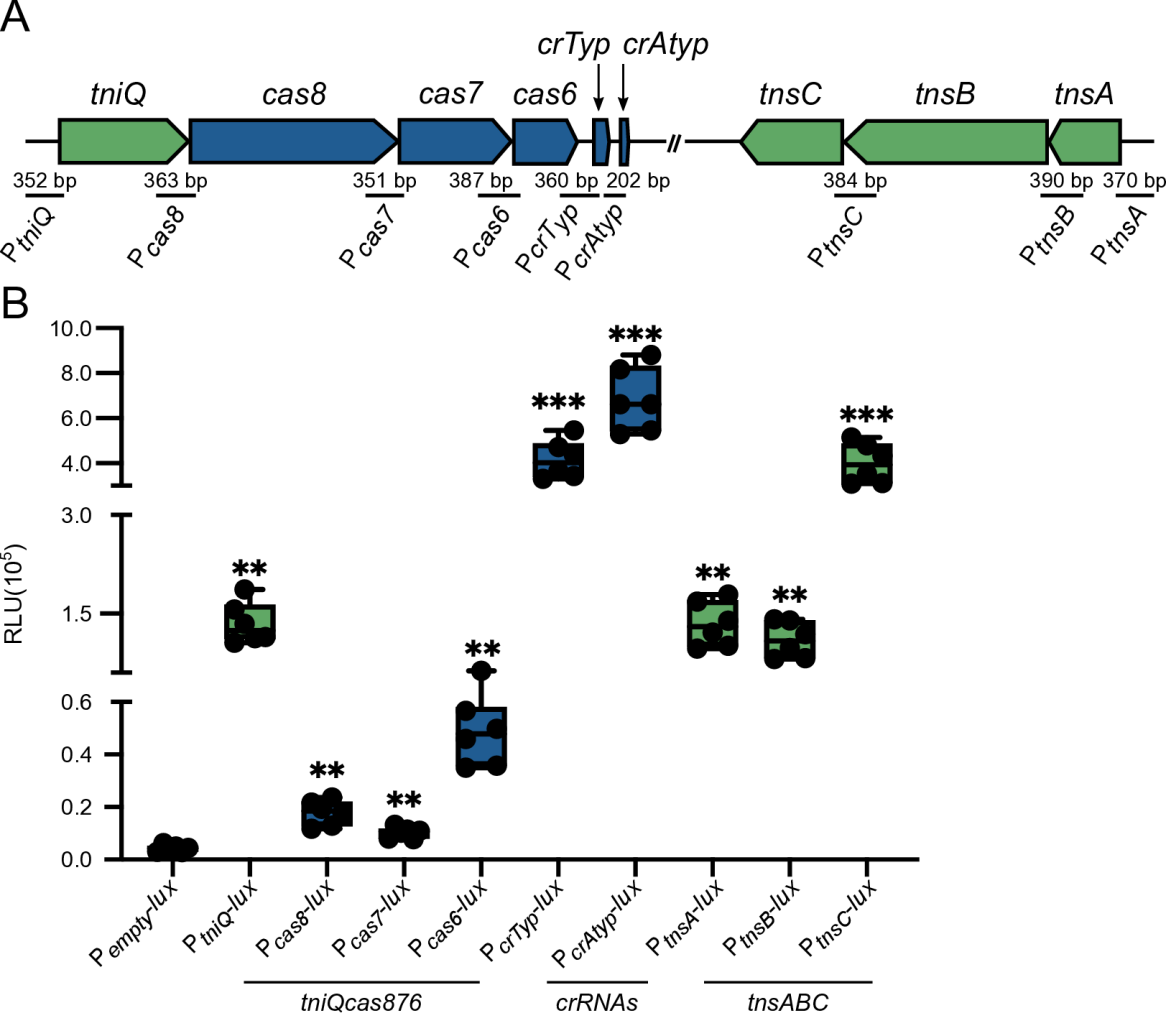
Multiple independent promoters drive transcription of genes associated with the *Vpa*CAST system. (**A**) Diagram illustrating the genetic arrangement of the components of the *Vpa*CAST system, and the regions used to generate each transcriptional fusion with the *luxCDABE* gene cluster. (**B**) Box plots represent the values of light production expressed as relative luminescence units (RLU) obtained from six independent biological replicates of strains harboring the transcriptional fusions of interest. The label P_empty_ refers to the negative control of a strain harboring the promoterless plasmid pBBRlux. Means were compared using a Brown-Forsythe and Welch ANOVA test followed by a Dunnett’s T3 test to compare each mean to the mean of the negative control (P_empty_). Mean differences with a *P* value ≤ 0.05 were interpreted as significant. ** Adjusted *P* value ≤ 0.01, *** ≤0.001.

To determine whether the genes that are part of the *V. parahaemolyticus* CRISPR/Cas-transposition system have independent promoters, we generated transcriptional fusions to the reporter *luxCDABE* gene cluster using the sequence upstream of each structural gene as well as the CRISPR arrays (Fig. 3A). The *luxCDABE* cluster encodes a bacterial luciferase and its substrate; thus, it can be used as a light-emitting genetic reporter. We used a strain that harbors the promoterless plasmid pBBRlux (P_empty_-*lux*) as a negative control. We were able to identify promoters with activity significantly different from the negative control upstream of *tniQ*, *cas8*, *cas7*, *cas6*, the typical and atypical CRISPR arrays, *tnsA*, *tnsB,* and *tnsC* (Fig. 3B). The transcriptional fusions with the upstream sequence from *cas7* and *cas8* showed low transcriptional activity with luminescence values close to those produced by the negative control; thus, more work needs to be done to assess whether these genes have independent promoters. The promoters upstream of *tniQ*, *tnsA,* and *tnsB* showed similar levels of activity. The most active promoters were those of the two CRISPR arrays and *tnsC*.

Our results suggest that the genes involved in CRISPR/Cas-mediated transposition are transcriptionally active under standard laboratory growth conditions and that the *tniQcas876* and *tnsABC* genetic clusters are likely composed of complex operons. We found several independent promoters with variable levels of activity that could potentially provide tunable regulation and/or contribute to the overall accumulation levels of the components of the *Vpa*CAST system.

### The promoters of *tniQ*, the atypical CRISPR array, VPA1391 (*vpxR*), and VPA1392 are subject to transcriptional regulation in *V. parahaemolyticus*

**Fig 4:**
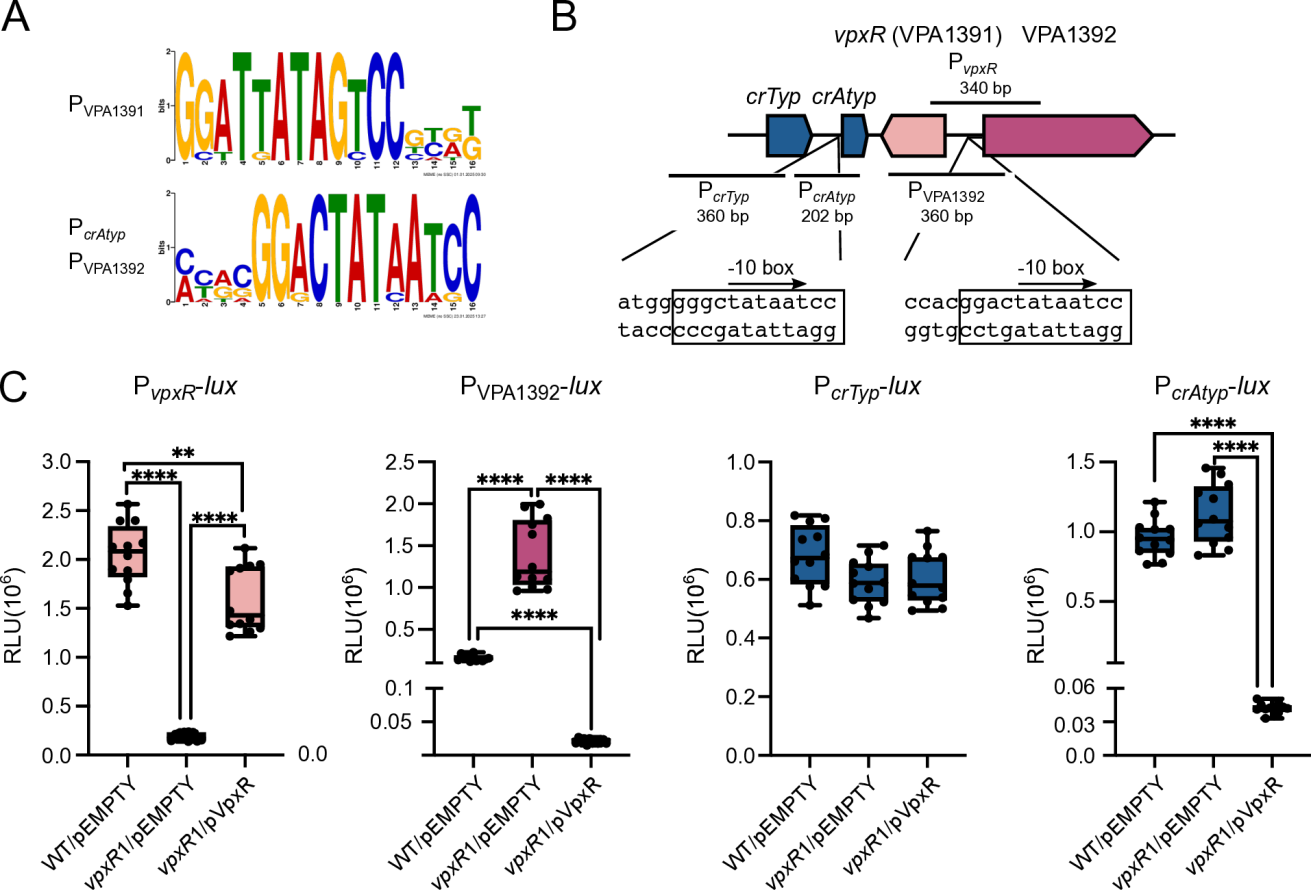
The VpxR (VPA1391) regulator positively regulates the P*vpxR* promoter and negatively regulates the P_VPA1392_ and P*_crAtyp_* promoters. (**A**) Logo (Forward and reverse complement orientation) generated with frequency matrices of a conserved motif discovered by the Multiple Em for Motif Elicitation software. (**B**) Diagram illustrating the arrangement of the CRISPR arrays and the *vpxR* and VPA1392 genes. The sequence of the VpxR-box and putative −10 boxes located upstream of the atypical CRISPR array and in the intergenic region between *vpxR* and VPA1392 is also shown. The putative VpxR-binding site is indicated with a rectangular box. (**C**) Box plots represent the values of light production expressed as relative luminescence units (RLU) obtained from 12 independent biological replicates from three independent experiments of strains harboring the transcriptional fusions of interest and either an empty overexpression plasmid (pEMPTY) or a plasmid that overexpresses *vpxR*. Means were compared using a Brown-Forsythe and Welch ANOVA test followed by a Dunnett’s T3 test for multiple comparisons. Mean differences with a *P* value ≤ 0.05 were interpreted as significant. **** *P* value ≤ 0.0001, ** *P* value ≤ 0.01.

Another fundamental question that we wanted to tackle in this work was whether the activity of the promoters that drive the expression of the elements of the *Vpa*CAST system was subject to regulation. The product of VPA1391, which is encoded downstream of the atypical CRISPR array, is a putative transcriptional regulator of the Xre family which has previously been shown to positively regulate its expression and negatively regulate the expression of the atypical CRISPR array from the *Vpa*CAST system. The evidence supporting these regulatory roles was obtained in the heterologous host *E. coli* and *in vitro* through biochemical experiments that demonstrated the interaction of VPA1391 with a specific DNA sequence (22). We used a similar bioinformatic approach as previously reported to locate the VPA1391-binding site (VPA1391-box) upstream of VPA1391 and the Atypical CRISPR matrix (P*_crAtyp_*) (Fig. 4A) (22). VPA1391 is encoded in the negative strand in a divergent orientation with respect to the gene VPA1392, which encodes a hypothetical protein (Fig. 4B). Since VPA1391 and VPA1392 share the same regulatory region, it is possible that both are regulated by the product of VPA1391 (Fig. 4B). The reported VPA1391-box overlaps with a putative −10 box element (TATAAT) from a putative promoter in both P*_crAtyp_* and P_VPA1392_ (Fig. 4B).

To evaluate the relevance of the regulatory role of VPA1391, here named *Vpa*CAST Xre-like regulator (VpxR), in its native genetic background, we first evaluated whether it has an active promoter and if it is autoregulated. We generated a P*_vpxR_-lux* transcriptional fusion, an insertion mutation in *vpxR* using *Vch*INTEGRATE (*vpxR*1) and a plasmid that overexpresses *vpxR* (pVpxR) through the activity of an IPTG-inducible promoter. Our results revealed that P*_vpxR_* is active in *V. parahaemolyticus* under standard laboratory growth conditions (Fig. 4C). The activity of P*_vpxR_* decreased significantly in a *vpxR*1 mutant strain compared to the WT strain, and the diminished activity of P*_vpxR_* in the *vpxR*1 mutant strain was complemented by expressing *vpxR in trans* (Fig. 4C). Together, these results strongly support that VpxR positively regulates its own transcription.

We next analyzed the effect of the absence or overexpression of *vpxR* on the activity of P_VPA1392_-*lux*, P*_crTyp_-lux,* and P*_crAtyp_-lux*. The absence of *vpxR* resulted in approximately a fivefold increase in the activity of P_VPA1392_ when compared to the activity in the WT strain (Fig. 4C). By contrast, the activity of P_VPA1392_ was approximately 24-fold higher in the WT strain compared to a strain that overproduces VpxR (Fig. 4C). These results strongly suggest that VpxR is a repressor of P_VPA1392_. Neither the absence nor the overexpression of *vpxR* affected the activity of P*_crTyp_* (Fig. 4C). This was somewhat expected since the regulatory region of P*_crTyp_* does not have a VpxR-box. Although the regulatory region of P*_crAtyp_* has a VpxR-box, we did not observe differences when comparing its activity in the WT strain and the *vpxR*1 mutant strain (Fig. 4C). However, the activity of P*_crAtyp_* in the WT strain was 17-fold higher than in a genetic background that overexpresses *vpxR* (Fig. 4C).

These results showed that VpxR is active in its native genetic background. The baseline production of VpxR under standard laboratory growth conditions is enough to activate P*_vpxR_* and repress P_VPA1392_. To observe the negative effect of VpxR on the activity of P*_crAtyp_*, it was necessary to overexpress it from an IPTG-inducible promoter.

**Fig 5:**
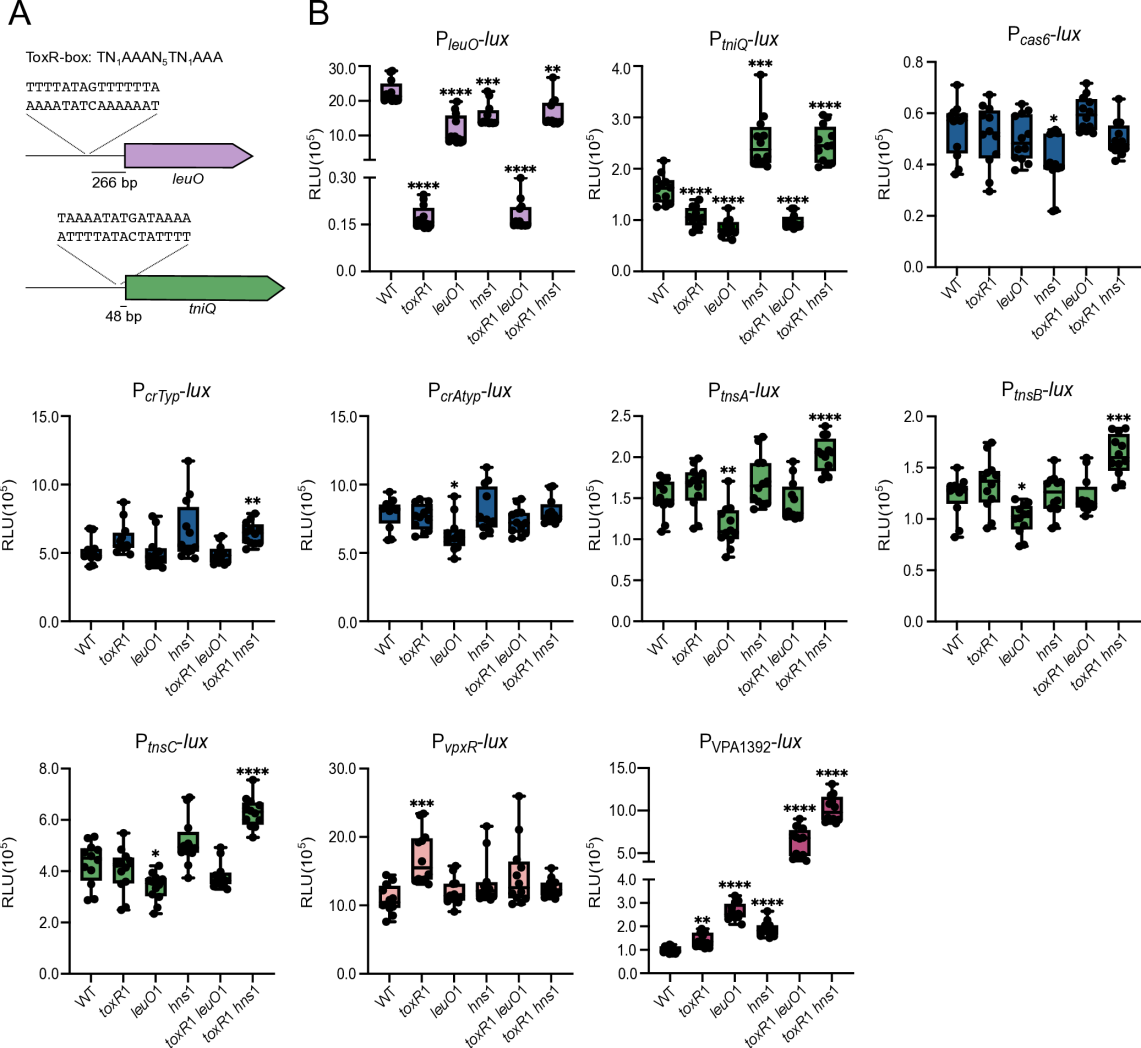
ToxR, LeuO, and H-NS modulate the expression of the P*_tniQ_* and P_VPA1392_ promoters. (**A**) Illustration depicting the sequence of the ToxR-box consensus and the putative ToxR-boxes located upstream of P*_leuO_* and P*_tniQ_* promoters. (**B**) Box plots represent the values of light production expressed as relative luminescence units (RLU) obtained from 12 independent biological replicates from three independent experiments of strains harboring the transcriptional fusions of interest. Means were compared using a Brown-Forsythe and Welch ANOVA test followed by a Dunnett’s T3 test for multiple comparisons. Mean differences with a *P* value ≤ 0.05 were interpreted as significant. **** *P* value ≤ 0.0001, *** *P* value ≤ 0.001, ** *P* value ≤ 0.01, * *P* value ≤ 0.05.

While analyzing a bioinformatic prediction of potential regulatory targets of ToxR in *V. parahaemolyticus,* we found that the regulatory region of *tniQ* had a consensus ToxR-box previously identified in ToxR-targets in *V. cholerae* (29) (Fig. 5A). This box is also present upstream of *leuO* on the reverse strand (Fig. 5A). LeuO, also named CalR in *V*. *parahaemolyticus*, is a transcriptional regulator of the LysR family whose transcription is positively regulated by ToxR in *V. cholerae* and *V. parahaemolyticus* (30–33). Based on these observations, we decided to evaluate whether the activity of P*_tniQ_* was affected by the absence of ToxR. We used *Vch*INTEGRATE to generate an insertion mutation in *toxR* (*toxR*1). As a control, we also analyzed the expression of a transcriptional fusion of P*_leuO_-luxCDABE*.

The activity of P*_leuO_* was 13-fold higher in the WT strain compared to that in the *toxR*1 mutant strain. The absence of ToxR had a modest negative effect on the activity of P*_tniQ_*, showing a fold change of 0.66 when compared to WT (Fig. 5B). ToxR and LeuO orthologs from multiple enterobacteria have been shown to antagonize the silencing effect of the histone-like nucleoid protein H-NS or coregulate H-NS targets (34–37). Based on this, we next evaluated whether null mutations in *leuO* and *hns* affected the activity of P*_leuO_* and P*_tniQ_*. We used *Vch*INTEGRATE to generate single mutants in *leuO* (*leuO*1) and *hns* (*hns*1) and double mutants in *toxR* and *leuO* (*toxR*1 *leuO*1) and *toxR* and *hns* (*toxR*1 *hns*1). The activity of P*_leuO_* decreased 0.5-fold in the mutant strains *leuO*1 and *hns*1 compared to that in the WT strain (Fig. 5B). The activity of P*_leuO_* in the *toxR*1 *leuO*1 double mutant strain was the same as in the *toxR*1 single mutant strain, while its activity in the *toxR*1 *hns*1 double mutant strain was the same as in the *hns1* single mutant strain (Fig. 5B). These results support a role for ToxR as an antagonist of H-NS at the P*_leuO_* promoter. The activity of P*_tniQ_* showed a fold change of 0.53 when comparing the *leuO1* mutant strain and the WT strain, and a 1.6 fold change in activity when comparing the *hns1* mutant strain and the WT strain (Fig. 5B). On one hand, the activity of P*_tniQ_* in the *toxR1 leuO1* double mutant strain was comparable to the activity in the single *toxR*1 and *leuO*1 mutant strains (Fig. 5B). And on the other hand, the activity of P*_tniQ_* in the *toxR1 hns*1 double mutant strain was the same as in the *hns*1 single mutant strain. These results suggest that ToxR and/or LeuO could antagonize the modest silencing activity of H-NS at the P*_tniQ_* promoter.

We also evaluated the effect of the absence of ToxR, LeuO, and H-NS on the activity of P*_cas6_,* P*_crTyp_,* P*_crAtyp_,* P*_tnsA_,* P*_tnsB_,* P*_tnsC_,* P_VPA1391_ and P_VPA1392_. The only promoter that showed fold changes in activity above 1.5 when comparing the WT, single, and double mutant strains was P_VPA1392_ (Fig. 5B). The activity of these promoters increased 2.6-fold in the *leuO* single mutant strain when compared to the WT strain. The combined absence of *leuO* and *toxR* or *toxR* and *hns* resulted in a 6-fold and 10-fold increase, respectively, in the activity of P_VPA1392_ when compared to the activity in the WT strain (Fig. 5B).

ToxR, LeuO, and H-NS appear to modulate the expression of P*_tniQ_* but do not seem to be strong regulators of the *Vpa*CAST system under our experimental conditions. ToxR, LeuO, and H-NS showed a significant silencing effect on the expression of VPA1392; however, it is not known whether this open reading frame forms part of the *Vpa*CAST system.

### A synthetic construct expressing the *Vpa*CAST system promotes programmable insertion of a mini-CAST with different efficiency in *E. coli* compared to *V. parahaemolyticus*

Based on transposition assays carried in the heterologous host *E. coli*, it has been previously suggested that the elements involved in CRISPR/Cas-mediated transposition from *V. parahaemolyticus* are functional (22, 23). Transposition mediated by the *Vpa*CAST system was found to be less efficient compared to transposition mediated by the *Vch*CAST system. To begin to investigate the potential roots of the differences in efficiency of these two CAST systems, we compared the degree of conservation at the amino acid level between the proteins from their Cascade and Transposase complexes using EMBOSS Needle (Table 2) (38). The TniQ, Cas8, Cas7, and Cas6 had % identities that go from 45% to 59%, while the TnsA, TnsB, and TnsC proteins had % identities that go from 30% to 41%. We also analyzed the conservation of putative TnsB-boxes (16, 26) within the L-terminal and R-terminal sequences that delimit the *Vch*CAST and the *Vpa*CAST (Fig. 1B). TnsB-boxes from the L-end were more conserved than the TnsB-boxes from the R-end (Fig. 1B). The direct repeats of the typical CRISPR array from *Vch*CAST and the *Vpa*CAST systems were highly conserved, while the imperfect repeats from the atypical CRISPR arrays were not (Fig. 1C).

**TABLE 2:** Pairwise comparison of amino acid identity between *Vpa*CASTm and *Vch*CASTm components

CASTm component	*Vpa*CASTm vs *Vch*CASTm % Identity
TniQ	57.8
Cas8	46.2
Cas7	55.4
Cas6	58.8
TnsA	29.8
TnsB	35.4
TnsC	40.8

**Fig 6:**
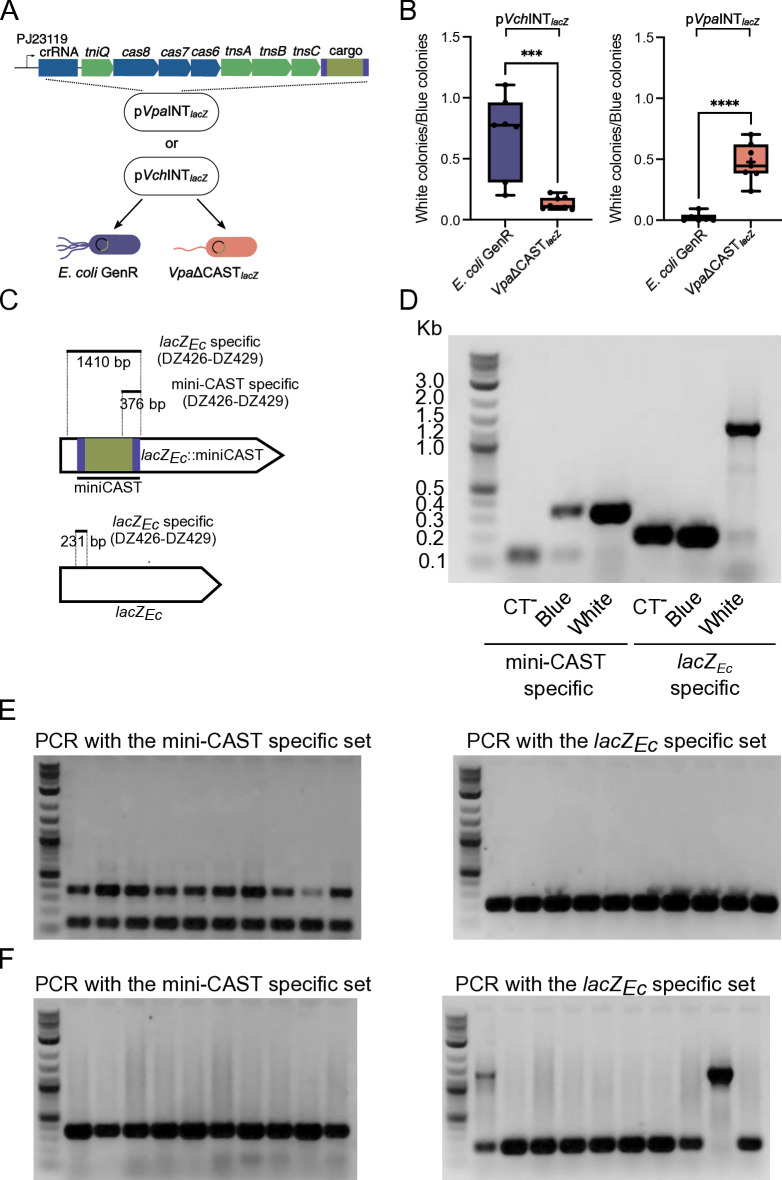
The CRISPR/Cas-transposition system from *V. parahaemolyticus* promotes transposition in *E. coli* and *V. parahaemolyticus*. (**A**) Diagram illustrating the screening assay used to analyze transposition events in the *lacZ_Ec_* allele from *E. coli* GenR and *Vpa*ΔCAST*_lacZ_* strains harboring either the p*Vch*INT*_lacZ_* or p*Vpa*INT*_lac_*_Z_ plasmid. (**B**) Box plots of the proportion of white colonies over blue colonies from *E. coli* GenR and *Vpa*ΔCAST*_lacZ_* strains harboring either the p*Vch*INT*_lacZ_* or p*Vpa*INT*_lac_*_Z_ plasmid grown in selection plates with X-gal and IPTG. Experiments were done at least six times. The differences between means were analyzed with an unpaired t-test. Mean differences with a *P* value ≤ 0.05 were interpreted as significant. **** indicate a *P* value ≤ 0.0001. ** indicate a *P* value ≤ 0.01. (**C**) Diagram of the regions amplified by PCR to identify transposition events and intact *lacZ_Ec_* copies. The solid lines represent the amplified regions and the region corresponding to the mini-CAST. (**D**) Image of the electrophoretic migration in an agarose gel of PCR products stained with ethidium bromide (EtBr) and obtained using as template the lysate of a blue colony from a *V. parahaemolyticus* strain harboring p*Vpa*INT (CT^-^), or blue and white colonies from a *V. parahaemolyticus* strain harboring p*Vpa*INT*_lacZ_*. (**E, F**) Image of the electrophoretic migration in an agarose gel of PCR products stained with EtBr and obtained using as template the lysate of 10 randomly selected colonies of (**E**) *E. coli* or (**F**) *V. parahaemolyticus* strains harboring p*Vpa*INT*_lacZ_*. All gels were loaded in the first lane with the Quick-Load Purple 1 kb Plus DNA Ladder from New England Biolabs. The relevant size range of the ladder is indicated on the gel from panel D.

It is not known if any of the above-mentioned differences are responsible for the variation in transposition efficiency mediated by these two CAST systems. We speculated that the *Vpa*CAST system may have adapted its function to the genetic context of *V*. *parahaemolyticus,* and this had concomitantly caused it to lose activity in other hosts. To begin to tackle this hypothesis, we analyzed *Vpa*CAST-mediated transposition in *E. coli* and *V. parahaemolyticus*. To do this, we followed a similar approach as previously reported for the generation of the gene editing tool INTEGRATE (26). This tool contains the elements required for CRISPR/Cas-mediated transposition present in the transposon Tn6677 from the *V. cholerae* HE-45 strain. These elements include the CRISPR/Cas-transposition machinery (CASTm) composed of TniQ, Cas8, Cas7, Cas6, TnsA, TnsB, and TnsC, a CRISPR matrix composed of two direct perfect repeats and a programmable spacer sequence, and a mini CRISPR-associated transposon (mini-CAST) flanked by right- and left-terminal sequences containing a non-symmetric arrangement of recognition sites for TnsB (Fig. 1B). All these elements are assembled on a pBBR1MCS-2 plasmid backbone that is named pSL1142, and we refer to here as *Vch*INTEGRATE. To generate a *V. parahaemolyticus*-based INTEGRATE plasmid (*Vpa*INTEGRATE), we replaced all the components corresponding to the CASTm, the CRISPR matrix, and mini-CAST in pSL1142 for sequences corresponding to the counterpart elements located in VpaI-7 in chromosome 2 of *V. parahaemolyticus* RIMD2210633 (Fig. 6A).

It has been documented that a CAST insertion event generates immunity toward new CAST transposition events (16, 26). Since the *V. parahaemolyticus* RIMD2210633 genome has all the elements of a CAST, we removed these elements to generate a genetic background that lacks the CASTm, CRISPR arrays, and the L- and R-terminal sequences that are recognized by TnsB. We will refer to this genetic background as ΔCAST from here on. To facilitate the detection of transposition events through genetic screenings, we generated a reporter strain with a ΔCAST, *lacZ_Ec_* genotype (*Vpa*ΔCAST*_lacZ_*). In contrast to the WT strain and the ΔCAST mutant strain, *Vpa*ΔCAST*_lacZ_* produces a functional LacZ enzyme (from *E*. *coli*) that is capable of degrading X-gal and thus allows screening for colonies with β-galactosidase activity based on blue versus white colony-coloration (Fig. 6A). We reprogrammed the CRISPR matrix from the *Vpa*INTEGRATE plasmid (p*Vpa*INT) with a 32 base pair sequence that encodes a crRNA that is complementary to *lacZ_Ec_* (p*Vpa*INT*_lacZ_*) (Fig. 6A). As a positive control, we also reprogrammed the *Vch*INTEGRATE plasmid (p*Vch*INT) with the same *lacZ_Ec_*-targeting crRNA to generate p*Vch*INT*_lacZ_*. As negative controls, we mobilized through conjugation the non-targeted plasmids p*Vch*INT and p*Vpa*INT to a derivative of *E. coli* BL21 DE3 that is gentamicin resistant (*E. coli* GenR) or to *Vpa*ΔCAST*_lacZ_*. All the transconjugant colonies showed a blue coloration when grown in the presence of X-gal (Data not shown). We next evaluated whether p*Vch*INT*_lacZ_* and p*Vpa*INT*_lacZ_* were able to promote transposition of the mini-CAST inside *lacZ_Ec_* in *E. coli* GenR and *Vpa*ΔCAST*_lacZ_*. The proportion of white vs blue colonies in transconjugants of *E. coli* GenR and *Vpa*ΔCAST*_lacZ_* with p*Vch*INT*_lacZ_* was on average 0.7 and 0.13, respectively (Fig. 6B). This would suggest that under the conditions tested, the *Vch*INTEGRATE system was more efficient in promoting transposition in *E. coli* GenR than in *Vpa*ΔCAST*_lacZ_*. By contrast, the proportion of white vs blue colonies in transconjugants of *E. coli* GenR and *Vpa*ΔCAST*_lacZ_* with p*Vpa*INT*_lacZ_* was, on average, 0.02 and 0.5, respectively (Fig. 6B). This would suggest that the *Vpa*INTEGRATE system is more efficient in promoting transposition in *Vpa*ΔCAST*_lacZ_* than in *E. coli* GenR. These results support previous observations that showed that the *Vch*CAST system was more efficient than the *Vpa*CAST system in promoting transposition in *E. coli*. Furthermore, our data suggest that host factors might affect the transposition efficiency mediated by CAST systems, but more work needs to be done to fully demonstrate that this is the case.

To further evaluate transposition events, we used an endpoint PCR-based approach that required two primer pairs. The first pair (DZ432-DZ429), here referred to as the mini-CAST specific set, only allows detection of transposition events (Fig. 6C). The second pair (DZ426-DZ429), here referred to as the *lacZ_Ec_* specific set, amplifies either a short sequence of 231 bp corresponding to an intact *lacZ_Ec_* allele or a 1,410 bp sequence corresponding to an insertion event of the mini-CAST in *lacZ_Ec_* (Fig. 6C).

We first used these primer sets in *Vpa*ΔCAST*_lacZ_* strains that received either p*Vpa*INT (negative control) or p*Vpa*INT*_lacZ_* and that were grown in screening plates containing X-gal and IPTG. When we used the lysate of a blue colony from the negative control strain as a template for amplification, as expected, the mini-CAST-specific primer set did not amplify the 376 bp amplicon corresponding to transposition events. We did observe a smaller unspecific band with a size between 100 and 200 bp (Fig. 6D). When we used a lysate of a blue colony of strain *Vpa*ΔCAST*_lacZ_*/p*Vpa*INT*_lacZ_* as a template for amplification with the miniCAST-specific primer set, we obtained both the specific 376 bp amplicon corresponding to transposition events and the unspecific amplicon observed in the negative control (Fig. 6D). This would suggest that even in blue colonies from strains harboring p*Vpa*INT*_lacZ_*, there might be cells with transposition events. When we used the lysate of a white colony of strain *Vpa*ΔCAST*_lacZ_*/p*Vpa*INT*_lacZ_* as template for PCR with the mini-CAST-specific primer set, we only obtained the 376 bp amplicon that is specific for transposition events in *lacZ_Ec_* (Fig. 6D). When we used the *lacZ_Ec_*-specific primer set, we were able to identify intact copies of *lacZ_Ec_* in the blue colonies of the negative control and the *Vpa*ΔCAST*_lacZ_*/p*Vpa*INT*_lacZ_* strain, and interrupted copies of *lacZ_Ec_* only in the white colony from the *Vpa*ΔCAST*_lacZ_*/p*Vpa*INT*_lacZ_* strain (Fig. 6D). We also observed a faint band that could correspond to intact copies of *lacZ_Ec_* in the white colony, suggesting that it may still have cells without a transposition event (Fig. 6D). Since the blue coloration of a colony suggests a majority of *lacZ*^+^ cells and the white coloration a majority of *lacZ*^-^ cells, our results suggest that the mini-CAST-specific primer set can detect rare transposition events within a colony, while the *lacZ_Ec_*-specific primer set can only detect transposition events when they occur at a high proportion within a colony.

We next used the two primer sets to analyze transposition events in 10 randomly selected colonies from *E. coli* GenR/p*Vpa*INT*_lacZ_* and *Vpa*ΔCAST*_lacZ_*/p*Vpa*INT*_lacZ_* strains grown in the absence of X-gal. The 10 colonies analyzed from strain *E. coli* GenR/p*Vpa*INT*_lacZ_* had transposition events that were revealed when we used the mini-CAST-specific primer set but not with the *lacZ_Ec_*-specific primer set (Fig. 2E). The 10 colonies analyzed from strain *Vpa*ΔCAST*_lacZ_*/p*Vpa*INT*_lacZ_* had transposition events that were revealed when we used the mini-CAST-specific primer set, and two of these colonies had transposition events that were also revealed when we used the *lacZ_Ec_*-specific primer set. Thus, the PCR results shown in Fig. 2E and F support our observation that transposition efficiency promoted by the *Vpa*INTEGRATE system is likely higher in the *V. parahaemolyticus* background compared to the *E. coli* background.

### The promoters that control the expression of the *Vpa*CAST system are more active in *V. parahaemolyticus* than in *E. coli*

**Fig 7:**
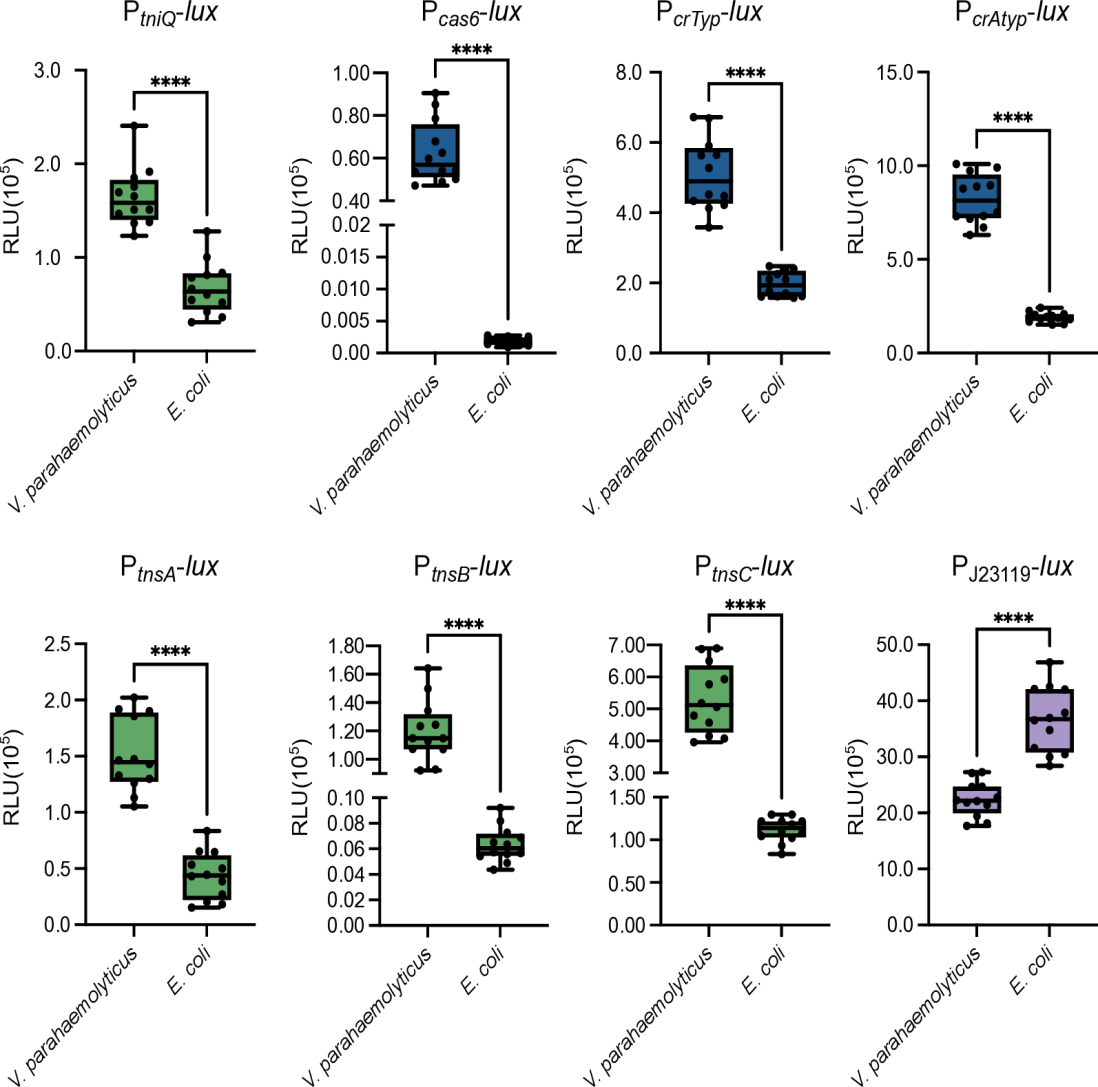
The promoters that drive transcription of genes associated with the *Vpa*CAST system are more active in *V. parahaemolyticus* than in *E. coli*. Box plots represent the values of light production expressed as relative luminescence units (RLU) obtained from 12 independent biological replicates of *V. parahaemolyticus* and *E. coli* strains harboring the transcriptional fusions of interest. The data were obtained from three independent experiments. Pairwise comparisons of means were done using an unpaired t-test. Mean differences with a *P* value ≤ 0.05 were interpreted as significant. **** *P* value ≤ 0.0001.

The p*Vpa*INT plasmid used in the experiments from Fig. 6 contains several of the internal promoters that we found to be transcriptionally active. We speculated that a possible explanation for the differences in the efficiency of the *Vpa*INTEGRATE plasmid in promoting transposition of a mini-CAST in an *E. coli* vs *V. parahaemolyticus* genetic background could be that these internal promoters are less active in *E. coli* than in *V. parahaemolyticus*. We compared the transcriptional activity in *V. parahaemolyticus* and *E. coli* of the active promoters identified in Fig. 3B and the synthetic promoter P_J23119_ used to drive the expression of the INTEGRATE tool. All the promoters from the genes of the *Vpa*CAST system were less active in *E. coli* than in *V. parahaemolyticus* (Fig. 7). By contrast, P_J23119_ showed a 1.6-fold increase in activity in *E. coli* compared to *V. parahaemolyticus* (Fig. 7). The P*_cas6_* promoter showed almost no activity in *E. coli*. The rest of the promoters showed between twofold and fourfold increased activity in *V. parahaemolyticus* compared to *E. coli*, except for the promoter P*_tnsB_* which showed approximately 19-fold higher expression in *V. parahaemolyticus* compared to *E. coli* (Fig. 7).

With these observations alone, we cannot conclusively affirm that the differences in transposition efficiency mediated by the *Vpa*CAST system in *V. parahaemolyticus* vs *E. coli* are due to changes in expression levels of its components, but we consider this should be a factor to be considered in future studies.

## DISCUSSION

The recently discovered CAST systems are an example of how the coevolution of mobile genetic elements (MGEs) and bacterial defense systems can result in the emergence of biological innovations. Although our understanding of the transposition capacity, structure, and regulation of different CASTs has progressively improved, we still have limited insight into how these MGEs operate in their native hosts (15, 23, 26, 39–44). Evaluating the behavior of CASTs in their native context could improve our understanding of their function, and learning how they are regulated and their impact on cell physiology could unveil biological principles of the mechanisms that govern the coevolution of these MGEs and their hosts. Here we report the first genetic characterization of the CAST I-F3a system from *V. parahaemolyticus* RIMD2210633, which is part of the VpaI-7 pathogenicity island and is possibly responsible for disseminating virulence factors (T3SS2 and TDH) between *Vibrio* strains in the environment. A key piece of information that could suggest that the *Vpa*CAST system is relevant for the physiology of *V. parahaemolyticus* RIMD2210633 is that of the transcriptional activity of its components. An *in silico* analysis of the nucleotidic and amino acid sequence of the *Vpa*CAST components suggests that they are somewhat conserved when compared to the components of the *Vch*CAST system. No clear indications of frame shifts or other deleterious mutations within the structural genes associated with the *Vpa*CAST system were found (23). However, mutations or sequence determinants that could affect their transcription are harder to identify by visual inspection. Here we showed that the *tniQcas876* and *tnsABC* genes are transcribed under standard laboratory growth conditions, they form polycistronic transcripts in *V. parahaemolyticus*, and have several internal promoters. The biological significance of these internal promoters needs to be further analyzed within their genetic context to determine how meaningful they are to the expression of the *Vpa*CAST system.

We found that the regulator VpxR (VPA1391) is expressed and controls the activity of the P*_vpxR_* and P*_vpa1392_* promoters in *V. parahaemolyticus*. Under the conditions tested, the overexpression but not the baseline production of VpxR repressed the transcriptional activity of the P*_crAtyp_* promoter. Finally, we showed that the regulators ToxR, LeuO, and H-NS modulate the expression of *tniQ*. We did not observe a clear effect of these regulators on the transcription of other genes from the VPA1387-VPA1396 region except for VPA1392. We cannot rule out that analyzing these regulatory regions outside of their native genetic context could result in topological changes that mask the potential effects of VpxR, ToxR, LeuO, and H-NS on their transcription. More work needs to be done to determine the physiological relevance of the regulation of these genes by the above-mentioned regulators and perhaps additional transcriptional regulators.

The implication of having an actively transcribed *Vpa*CAST system is still unclear. We still do not know whether the transcription levels of the *Vpa*CAST system observed in this study are sufficient to promote transposition, and we are currently working on tackling this question. It would seem wasteful to have unregulated production of an MGE that can only be mobilized to specific targets that might not be always available inside the cell. However, a constitutive expression of the components of the *Vpa*CAST system is perhaps necessary for an efficient transposition toward plasmids acquired through conjugation or natural competency. The typical CRISPR array located in the VpaI-7 island has two spacers, one of which targets plasmids such as pL300 that can be hosted by *V. alginolyticus* strains (24, 25). It will be of great importance to determine whether the constitutive expression of the typical crRNA from the *Vpa*CAST system is sufficient to promote transposition into plasmids of ecological relevance. The regulation of the *Vpa*CAST system, at the transcriptional level and beyond, needs to be further studied, as well as the relevance of its baseline level of expression for yet unidentified physiological roles.

Previous studies performed in the heterologous host *E. coli* showed that the Xre regulator encoded in VpaI-7, here referred to as VpxR, acts as a repressor of the activity of the P*_crAtyp_* promoter (22), in addition to autoregulating its own expression through a mechanism similar to that of the repressor protein CI from phage lambda (45–47). The strong repression exerted by VpxR against P*_crAtyp_* led the authors to propose that CAST mobility is negatively regulated at the transcriptional level when the CAST has been established inside a host (22). However, our findings suggest that the atypical CRISPR array and *vpxR* are expressed under standard laboratory growth conditions and that the expression of the former is not affected by the absence of the latter. The repressive nature of VpxR over the activity of the P*_crAty_* promoter was only observed when the regulator was artificially overexpressed. We did observe the autoregulation of *vpxR* and found that it negatively regulates the divergent gene VPA1392, which encodes a hypothetical protein. The VpxR-box found upstream of P*_crAty_* and P_VPA1392_ has some differences that could possibly affect the strength of the interaction of VpxR with each of these two regulatory regions. Our group is currently focused on the characterization of these regulatory regions and on analyzing whether the role of VpxR goes beyond the control of the expression of the atypical CRISPR array from VpaI-7.

Another key observation that casts some doubts on the functionality of the *Vpa*CAST system is its reduced ability to promote the transposition of synthetic mini-CASTs in *E. coli* when compared to the ability of the *Vch*CAST system (23). These differences in functionality could be due to several factors. An interesting observation is that of the apparent separate evolutionary trajectories of the *tniQcas876* and *tnsABC* operons even among closely related bacteria (13, 23). Apparently, the components of the *tniQcas876* operon are subject to evolutionary forces that result in higher conservation at the DNA and protein level, while the components of the *tnsABC* system appear to be more divergent. To our knowledge, there has not been a clear association with divergency and changes in functionality other than target categorization to avoid cross-talk among related transposition systems, but they could potentially explain why some CAST systems are more efficient than others in promoting transposition (22, 23). The divergence in TnsA, TnsB, and TnsC is also accompanied by divergence in the TnsB-boxes that are recognized by the transposase complex (23, 44). While the three TnsB-boxes present in the L-terminal sequence of the *Vpa*CAST closely resemble the consensus CA[A/C]CCATA[A/T] [A/G]NTGATA[T/A][T/C][T/G] of TnsB-boxes present in *Vch*CAST systems, the TnsB-boxes from the R-terminal sequence significantly diverged (23, 44). It is not exactly known how these deviations from the consensus in the TnsB-boxes affect the affinity of TnsB for the R-terminal sequence. As mentioned above, it has been suggested that these sequence changes were selected to avoid cross-talk between CAST systems, it would be interesting to evaluate in the future whether loss of efficiency can be compensated by the emergence of novel *in trans* or *cis* regulatory mechanisms. In this study, we provided evidence that suggests that the transposition efficiency of the *Vpa*CAST system is higher in its native host, *V. parahaemolyticus*, than in *E. coli*. The ramifications of this observation need to be studied further to better understand the conditions and factors that can lead to differences in transposition efficiency in different genetic backgrounds. We observed that the internal promoters within the *tniQcas876* and *tnsABC* operons were more active in *V. parahaemolyticus* than in *E. coli*, so perhaps they accumulate to higher concentrations in the former. The threshold of *Vpa*CASTm accumulation needed to achieve maximal transposition efficiency is unknown and will be important to define in the future. Beyond the transcriptional-regulation explanation, it is tempting to speculate that “host factors” could intervene to facilitate the transposition process in genetic backgrounds where these MGEs have evolved. Such host factors have been reported and include ACP and L29 that favor Tn7 transposition in *E. coli* (48), IHF and ClpX, which enhance transposition efficiency of the *Vch*CAST system (44, 49), and the S15 ribosomal subunit that does so for the CRISPR-associated transposition system of *Scytonema hofmannii* (*Sho*CAST system) (50, 51). The identification of potential “*Vibrio* integration factors” would be of great relevance to understand the reach and limitations of CASTs in shaping the evolution of *Vibrio* species. Furthermore, the identification of such factors could also help to broaden the host range of *Vch*CAST- and *Vpa*CAST-based engineering tools.

CRISPR-associated transposition appears to have played an important role in the evolution of a variety of bacterial species, and the fact that the components of the CASTm encoded in several genomes retain functionality opens the question of what their active contribution to horizontal gene transfer in the environment is. Here we reported our initial findings on the characterization of a CAST system that could have played an important role in the emergence of pandemic attributes in *V. parahaemolyticus*. Based on the potential that these MGEs have to propagate virulence factors, we consider it of great relevance to continue the study of the factors that limit or favor their capacity for horizontal gene transfer. A better understanding of the functionality of the CAST systems in their native host could inform us how they might have shaped or could actively be shaping the evolution of human pathogens such as *V. parahaemolyticus*.

## MATERIALS AND METHODS

### Strains, plasmids, and growth conditions

The strains and plasmids used in this study are described in Table 3. All strains were grown in Lysogeny Broth (LB) (1% tryptone, 0.5% yeast extract, and 1% NaCl) or on LB agar (LB with 1.5% bacteriological agar). Strains derived from *V. parahaemolyticus* RIMD2210633 were grown at 30°C and *E. coli* strains were grown at 37°C. Strains and plasmids were selected by adding antibiotics to the growth media at the following concentrations: streptomycin (Str) at 200 µg/mL, kanamycin (Kan) at 30 µg/mL, gentamicin (Gen) at 15 µg/mL, chloramphenicol (Chl) at 20 µg/mL for *E. coli,* and 5 µg/mL for *V. parahaemolyticus*.

**TABLE 3:** Strains and plasmids used in this study

Strains	Genotype	Source reference
*E. coli*		
DZEc363 (BL21 (DE3))	*fhuA*2 [*lon*] *ompT gal* (λ DE3) [*dcm*] ∆*hsdS*	Unknown origin
DZEc1067 (*E. coli* GenR)	DZEc363 with an unmapped insertion of a mini Tn5 transposon with a gentamicin resistance marker. Gen^R^	This work.
DZEc345 (DH5α)	F^–^ φ80*lacZ*ΔM15 Δ(*lacZYA-argF*)U169 *recA*1 *endA*1 *hsdR*17(rK–, mK+) *phoA* s*upE*44 λ^–^*thi*-1 *gyrA*96 *relA*1	Unknown origin
DZEc104 (SY327 (*λpir*))	Δ(*lac pro*) *argE*(Am) *rif nalA recA56 λpir*	Unknown origin
DZEc344 (SM10 (*λpir*))	*thi, thr, leu, tonA, lacY, supE, recA,* RP4-2- Tc::Mu *λpir*, Kan^R^	Unknown origin
*V. parahaemolyticus*		
DZVp42	*Vibrio parahaemolyticus* O3:K6 RIMD2210633, Str^R^, Amp^R^	(52)
DZVp784	DZVp42, P*tniQ*::FRT-*aacC1-* VPA1397::sfGFP- FRT, Δ*lacZ*_Vp_::P_tac_-*lacZ*_Ec,_ Str^R^, Amp^R^, Gen^R^	This work
DZVp835 (*Vpa*ΔCAST*_lacZ_*)	DZVp42, ΔLeft end (L) CAST, Δ*tniQ-* VPA1397 (ΔCAST), Δ*lacZ*_Vp_::P_tac_-*lacZ*_Ec_	This work
DZVp1279	DZVp42, *vpxR1* (*vpxR*::miniCAST)	This work
DZVp949	DZVp42, *toxR*1 (*toxR*::miniCAST)	This work
DZVp1437	DZVp42, *leuO*1 (*leuO*::miniCAST)	This work
DZVp1439	DZVp42, *hns*1 (*hns*::miniCAST)	This work
DZVp1545	DZVp42, *toxR*1, *leuO*1	This work
DZVp1547	DZVp42, *toxR*1, *hns*1	This work
DZVp1637	DZVp42, *hns*1, *leuO*1	This work

Plasmids	Description	Source reference
pDZ387 (p*Vch*INT)	pSL1142-*Vch*INTEGRATE. *Vch*CAST from Tn6677 cloned into pBBRMCS backbone, Kan^R^.	Addgene plasmid # 160730 (26)
pJET 1.2 Blunt (pJET)	Blunt cloning of PCR products, Amp^R^.	Thermo Scientific
pDZ535	pJET-VcCRISPR-NT. CRISPR typical matrix from pDZ387 cloned into pJET by blunt ligation.	This work
pDZ583	pJET-VctnsABC. *tnsABC* from pDZ387 cloned into pJET.	This work
pDZ551	pJET-VcCargo-ChlR. miniCAST from pDZ387 cloned into pJET.	This Work
pDZ633	pJET-VpCRISPR-NT. CRISPR typical matrix from DZVp42 cloned into pJET.	This work
pDZ653	pJET-VpCRISPR-VptniQCascade. VpCRISPR typical matrix and *tniQ-cas876* from DZVp42 cloned into pJET.	This work
pDZ724	pJET-VpCRISPR- VptniQCascade_Ser231tca. VpCRISPR typical matrix and *tniQ-cas876* with a mutation *tniQ*-Ser231tca from DZVp42 cloned into pJET.	This work
pDZ744	pJET-VptnsABC. *tnsABC* from DZVp42 cloned into pJET.	This work
pDZ661	pJET-VpCargo-ChlR. miniCAST from DZVp42 cloned into pJET.	This work
pDZ681	pSL1142-VctnsABC-VpCargo. miniCAST from pDZ661 cloned into pDZ387.	This work
pDZ799	pSL1142-VptnsABC-VpCargo. *tnsABC* from pDZ744 cloned into pDZ681.	This work
pDZ879 (p*Vpa*INT)	pSL1142-VpaINTREGRATE. CRISPR and *tniQ*Ser231-*cas876* from pDZ724 cloned into pDZ681.	This work
pDZ952 (p*Vch*INT*_lacZ_*)	pSL1142-VchINT-crRNA_*lacZ*. *Vch*INTREGRATE with crRNA-*lacZ*.	This work
pDZ1000 (p*Vpa*INT*_lacZ_*)	pSL1142-VpaINT-crRNA_*lacZ*. *Vpa*INTREGRATE with crRNA-*lacZ*.	This work
pDZ63	pRE118. oriT R6K ori *sacB*1, *aphA*, Kan^R^	(53)
pDZ510	pMMMB67EH-Gen-*lacZ_Ec_*	This work
pDZ260	pRE118-FRT-KanR-FRT.	This work
pDZ518	pRE118-*lacZ*Ec.	This work
pDZ688	pRE118-*tniQ*::GenR-FRT. Kan^R^, Gen^R^.	This work
pDZ585	pRE118-VPA1397::GFP-FRT. Kan^R^	This work
pDZ759	pRE118-LeftEnd_Del. Kan^R^	This work
pDZ917	pRE118-LeftEnd-VpaI-GenR-FRT. Kan^R^, Gen^R^.	This work
pDZ514	pBBR5-flp-sacB. Gen^R^.	This work
pDZ527	pBBR2-flp-sacB. Kan^R^.	This work
pFY_1554	pBBRlux. Chl^R^.	(54)
pDZ689	pBBRlux-P*_tniQ_*	This work
pDZ691	pBBRlux-P*_cas8_*	This work
pDZ693	pBBRlux-P*_cas7_*	This work
pDZ695	pBBRlux-P*_cas6_*	This work
pDZ697	pBBRlux-P_crTypical_	This work
pDZ699	pBBRlux-P_crAtypical_	This work
pDZ701	pBBRlux-P*_tnsA_*	This work
pDZ703	pBBRlux-P*_tnsB_*	This work
pDZ705	pBBRlux-P*_tnsC_*	This work
pDZ892	pBBRlux-P*_vpa1391_*	This work
pDZ894	pBBRlux-P*_vpa1392_*	This work
pDZ728	pBBRlux-P_J23119_	This work
pDZ1028	pBBRlux-P*_toxR_*	This work
pDZ749	pBBRlux-P*_leuO_*	This work
pDZ931	pBBRlux-P*_hns_*	This work
pDZ782	pSL1142-crRNA_*toxR*.	This work
pDZ1068	pSL1142-crRNA_*leuO*.	This work
pDZ1070	pSL1142-crRNA_*hns*.	This work
pDZ1013	pSL1142-crRNA_VPA1391.	This work
pDZ9 (pFY_1554)	pMMB67EH-Gm. Expression vector harboring *lacI* and containing a Ptac promoter, Gen^R^.	Miller, F. Yildiz
pDZ913	pMMB-VPA1391. VPA1391 cloned into pDZ9.	This work
pDZ738	pBAMD1-6. Plasmid carrying a mini Tn5 transposon with a Gen^R^ cassette.	(55)

### RNA purification and cDNA synthesis

RNA was extracted from cells from strains DZVp42 (WT) and DZVp784 (P*_tniQ_*::FRT-*aacC1*) grown to the exponential phase in LB broth at 30°C with agitation, using the commercial kit Direct-zol RNA MiniPrep from Zymo Research following the instructions of the manufacturer. The total RNA was concentrated and treated with DNase I using the commercial kit RNA Clean and Concentrator-5 from Zymo Research. The complete removal of DNA from the extractions was verified through PCR.

The DNAase I-treated RNA was used as a template to synthesize cDNA with the commercial reagent LunaScript RT SuperMix following the manufacturer’s instructions.

The RT-PCR semiquantitative analysis was done from three independent experiments. The densitometric analysis of DNA bands stained with ethidium bromide was done using the Volume tools of the analysis software Image Lab from BioRad.

### Generation of genetic constructs

The primers used for the generation of genetic constructs are described in Table 4. Amplicons used for assembling genetic constructs were produced by PCR with the high-fidelity DNA polymerase Q5 from New England Biolabs. PCR purification and plasmid isolation were done using the DNA Clean & Concentrator-5 and Zyppy Plasmid Miniprep Kits from Zymo Research, respectively. Sanger sequencing was used to validate the fidelity of the genetic constructs generated for this study.

**TABLE 4:** Primers used in this study

Primer	Sequence 5′−3′	Construct/amplicon
Generation of pSL1142-*Vpa*INTEGRATE		
DZ406	GTCAGTGAGCGAGGACACAA	pDZ535
DZ407	GCTTCTAGGAAGCGATGGAC	pDZ535
DZ460	GGGCCCCCTTTAATAAGGAGATATACCATGGC GA	pDZ583
DZ430	CTGAAGTTTAGACCATGAAGAGGC	pDZ583
DZ469	TCCTTTTGAGGAGGGTGTTG	pDZ551
DZ35	GTAAAACGACGGCCAGT	pDZ551
DZ501	CTGATAATGAGACCTCTGTCTTGTCAGC	pDZ633
DZ502	CTACCTATTCGGCAGTTCACCCATGG	pDZ633
DZ503	CTGATAATGGATCCGAATTCGAGCGAAG	pDZ633
DZ504	CTACCTATTCGGCAGTTCACGAGACC	pDZ633
DZ538	ATCTTTCTAGAAGATCTCCTAC	pDZ653
DZ534	CATATGTATATCTCCTTCGCTC	pDZ653
DZ535	GCGAAGGAGATATACATATGGTTCAAATGATGC TTTTACAAC	pDZ653
DZ539	AGGAGATCTTCTAGAAAGATGGGCCCTTAAATC AAAGGAACAACCTG	pDZ653
DZ600	GCATTGGTGGTCACAAATATCAAG	pDZ724
DZ601	ATGAACCCCCATCTATAAC	pDZ724
DZ521	CCTAGGCTGCTGCGCTGC	pDZ744
DZ522	GGTATATCTCCTTATTAAAGGGGGCCC	pDZ744
DZ548	GTGTACCATACTTTCGAAAG	pDZ744
DZ549	TCAATCTCCTTTTGCAGC	pDZ744
DZ525	CTCGAGGTCATTTCCGGG	pDZ661
DZ526	ACAAATGCCTGAGGTTTCAG	pDZ661
DZ527	GAGTTTGTAAATACAACCATAC	pDZ661
DZ528	TTGTAGTAAACTTTCGAAAGTATG	pDZ661
DZ529	GGTACCGAGCTCGAATTC	pDZ661
DZ530	CTGCAGTAAGGACTCTAG	pDZ661
DZ531	GATACCTCAAATATATCGTCAC	pDZ661
DZ532	AACTCTGTTGATACAACC	pDZ661
Analysis of CRISPR/Cas-mediated transposition into *lacZ_Ec_*		
DZ426	CATCTACACCAACGTGACCTATCC	*lacZ_Ec_* 231 bp *lacZ_Ec_*::mini-CAST 1,410 bp
DZ429	GCGCTCAGGTCAAATTCAGAC	*lacZ_Ec_* 231 bp *lacZ_Ec_*::mini-CAST 1,410 bp
DZ432	CCATGTCGGCAGAATGCTTA	*lacZ_Ec_*::mini-CAST 376 bp
Analysis of gene transcripts through RT-PCR		
DZ602	GTCAACAACCGCGTTAATCC	*tniQ* 335 bp
DZ540	GGACATCTGGCAATGGTTTT	*tniQ* 335 bp
DZ711	ACTCCCATTGAAGCGCTAAC	*cas8* 341 bp
DZ710	CAATGAACGTCACCATTCCA	*cas8* 341 bp
DZ810	ACGGTAAACTGGCGAACCTT	*tniQ-cas8* 541 bp
DZ811	GCACGAATTACGCCATTTGC	*tniQ-cas8* 541 bp
DZ712	CGAAATCGACTTTTGCTGTG	*cas7* 338 bp
DZ542	TGAAGTGGTTAGTGCCAGCAT	*cas7* 338 bp
DZ541	ATGCAATCAAAGCGATACCC	*cas8-cas7* 539 bp
DZ818	TCGATTTCGTGGGGGTTACC	*cas8-cas7* 539 bp
DZ543	AACGGTGGGACGTAAAATCA	*cas6* 339 bp
DZ714	CGGAACTTATTGCCACTGCT	*cas6* 339 bp *cas7-cas6* 822 bp
DZ713	CCGCATCTATCAATCCACTG	*cas7-cas6* 822 bp
DZ716	TTGGACAGTGCCAATCAAAC	*tnsA* 192 bp
DZ717	TCATTCATCGCTACGCTTGT	*tnsA* 192 bp *tnsA-tnsB* 318 bp
DZ544	CTTGCAGTTCAATCCCGAAT	*tnsA-tnsB* 318 bp
DZ718	TTGGTTTCTATCTTGGGTTCG	*tnsB* 816 bp
DZ719	TTCGTGCATAATCGCAACAC	*tnsB* 816 bp
DZ545	CTGAGCGGACCAAATCTCAT	*tnsB-tnsC* 377 bp
DZ761	CGGGTAACGAAGTGAACAAAA	*tnsB-tnsC* 377 bp
DZ546	CGAATTAACCGCATGTTTCC	*tnsC* 354 bp
DZ547	CATTATCATAGATGAGTTCCAGCA	*tnsC* 354 bp
DZ808	GGCAATTCTGAGCTGCAATA	*rpoB* 842 bp
DZ809	CCGTGGTCTAGATCGTTCGT	*rpoB* 842 bp
Generation of constructs used to delete and insert sequences through homologous recombination		
DZ473	CAAGCTTCTTCTAGAGGTACCGACAGCATCAA CAGCAG	pDZ688
DZ489	TCGTCTGGATACTAGTCACCAACTTGATAGGCA C	pDZ688
DZ510	ACTAGTGATCGAATTGGGGATCTTG	pDZ688
DZ537	ACTAGTCATAAGCCTGTTCGGTTC	pDZ688
DZ490	GGTGACTAGTATCCAGACGAAAGCCTTG	pDZ688
DZ509	CATGAATTCCCGGGAGAGCTCGCGGATATATTT TCCCCAC	pDZ688
DZ479	CAAGCTTCTTCTAGAGGTACCAGAACAAGCGT AGCGATG	pDZ585
DZ480	TGATTCCCTTTGAATCAAAACACCTATACCTTG	pDZ585
DZ481	TTTTGATTCAAAGGGAATCAGGGGATCTTG	pDZ585
DZ482	ACGCGGCTAAGGCCTTCGCGAGGTACTTATTT G	pDZ585
DZ483	TTTTGATTCAAAGGGAATCAGGGGATCTTG	pDZ585
DZ484	ACGCGGCTAAGGCCTTCGCGAGGTACTTATTT G	pDZ585
DZ594	CAAGCTTCTTCTAGAGGTACCAAGTGATGCCTT TACATG	pDZ759
DZ595	TTCGGTCATAATTGTTCTCCCAATGTAG	pDZ759
DZ596	GGAGAACAATTATGACCGAATCGCTTGG	pDZ759
DZ597	CATGAATTCCCGGGAGAGCTGAGCTCTATAAC AAGTTGCTGCACAC	pDZ759
DZ389	CTATAGGGCGAATTGGAGCTGCATAGTGCGTG TTTATG	pDZ527
DZ390	CGATATCCTATAATTCGATGAAGATTCTTGC	pDZ527
DZ391	CATCGAATTATAGGATATCGGCATTTTCTTTTG	pDZ527
DZ392	AGGGAACAAAAGCTGGGTACATCACATATACC TGCCGTTC	pDZ527
Generation of transcriptional fusions constructs		
DZ576	CTATAGGGCGAATTGGAGCTCTTTGACTGCTTG ATTCG	pDZ689
DZ577	ATTTGCGGCCGCAACTAGAGCTTTCGTCTGGA TAAGATTTTG	pDZ689
DZ578	CTATAGGGCGAATTGGAGCTCGTTCGAACATC ATATTAAAAG	pDZ691
DZ579	ATTTGCGGCCGCAACTAGAGGCGCAAATCTTT ATTAAGG	pDZ691
DZ580	CTATAGGGCGAATTGGAGCTCCGTTGAAACAG GTAGATG	pDZ693
DZ581	ATTTGCGGCCGCAACTAGAGATCAGAAGCCTC AATAGATTTC	pDZ693
DZ582	CTATAGGGCGAATTGGAGCTCTGACGTAGATG GAGAGAG	pDZ695
DZ583	ATTTGCGGCCGCAACTAGAGATTTAGCTGCGA TTGCTTC	pDZ695
DZ584	CTATAGGGCGAATTGGAGCTTCATGCATTCCAA AGCTAC	pDZ697
DZ585	ATTTGCGGCCGCAACTAGAGCTGTTAGCATCT GCTTGAG	pDZ697
DZ586	CTATAGGGCGAATTGGAGCTAGTCGGTCAACT GAGAATAC	pDZ699
DZ587	ATTTGCGGCCGCAACTAGAGGCTTTTTGGCTG AGCCTG	pDZ699
DZ588	CTATAGGGCGAATTGGAGCTGTTGCCAACATG AATAGG	pDZ701
DZ589	ATTTGCGGCCGCAACTAGAGTTCATACAAAGTA TCCACAC	pDZ701
DZ590	CTATAGGGCGAATTGGAGCTGATGCACAGAAA CAACAAG	pDZ703
DZ591	ATTTGCGGCCGCAACTAGAGCCGAAGTATTCG AGTGTTTC	pDZ703
DZ592	CTATAGGGCGAATTGGAGCTAAAGTGTGTTGCG ATTATG	pDZ705
DZ593	ATTTGCGGCCGCAACTAGAGCCGAGAGAATTT CATCTATC	pDZ705
DZ691	TTGGAGCTCCCCTTGCTTTACCATTGTATTG	pDZ892
DZ692	AGAGGATCCTTTCGCATAGAACGAACG	pDZ892
DZ693	TTGGAGCTCCTGAAGGACACTTGCTATC	pDZ894
DZ694	AGAGGATCCTAAACGTGGGTTTGTTATTTATG	pDZ894
DZ603	CTATAGGGCGAATTGGAGCTTTTTATGCATGCG CCCAATAC	pDZ728
DZ604	ATTTGCGGCCGCAACTAGAGATGGTATATCTCC ACGTCG	pDZ728
DZ740	CTATAGGGCGAATTGGAGCTTCATTGCGGCTC ATACCG	pDZ1028
DZ767	ATTTGCGGCCGCAACTAGAGCAACATCAGGA GTATACGG	pDZ1028
DZ618	AGAGAGCTCTCCGTTGGTTATTGATAGGTGGG	pDZ749
DZ619	AGAGGATCCCATACGGTAGCTAGCAATTGC	pDZ749
DZ736	CTATAGGGCGAATTGGAGCTTACCGTACACATT ACAGC	pDZ931
DZ737	ATTTGCGGCCGCAACTAGAGCTAGAGCTTCTT CCAATTG	pDZ931
Generation of constructs used for transposon insertion-directed mutagenesis		
DZ656	ATAAAGACGCGAATTATTTTTGATGGCGTTAACT CG	pDZ1000
DZ657	TTCACGAGTTAACGCCATCAAAAATAATTCGCG TCT	pDZ1000
DZ624	ATAATGATGTTGGCGGAGAGACCAAACGAAGTT TTA	pDZ782
DZ625	TTCATAAAACTTCGTTTGGTCTCTCCGCCAACAT CA	pDZ782
DZ776	ATAACGTATGGAAAGCACTTTGCGTGGTGTAGA TTTGG	pDZ1068
DZ777	TTCACCAAATCTACACCACGCAAAGTGCTTTCC ATACG	pDZ1068
DZ780	ATAACGTAGCCTTCGTGCATTTTCTCGTGAGCT GACTG	pDZ1070
DZ781	TTCACAGTCAGCTCACGAGAAAATGCACGAAG GCTACG	pDZ1070
DZ753	ATAACTACGTAGCTACGATCTATATCTGCAGCC AAAGG	pDZ1013
DZ754	TTCACCTTTGGCTGCAGATATAGATCGTAGCTA CGTAG	pDZ1013
DZ755	ATAACAGCACGATGCAAGACAAAAGTGTAGGTT ATGTG	pDZ1015
DZ756	TTCACACATAACCTACACTTTTGTCTTGCATCGT GCTG	pDZ1015
Generation of constructs for gene overexpression		
DZ730	CAGGAAACAGAATTCGAGCTATGAGAGACTTA GCAAAAAAG	pDZ913
DZ731	CCTGCAGGTCGACTCTAGAGTTATGGTAGAAG CTCTCG	pDZ913

The transcriptional fusions used in this study were generated by cloning upstream sequences from the genes of interest into the pBBRlux plasmid linearized with the restriction enzymes SacI-HF and BamHI-HF (New England Biolabs). Cloning was done either by isothermal assembly using the NEBuilder HiFi DNA Assembly Master Mix or by ligation with a T4 DNA ligase.

The construct for the overexpression of *vpxR* was generated in the backbone of the plasmid pMMB67EH-Gen. The *vpxR* coding sequence was cloned downstream of the Ptac promoter from pMMB67EH-Gen through an isothermal assembly.

The plasmid containing the *Vpa*INTEGRATE system (pDZ879) was generated from intermediary constructs and assembled sequentially in a modular fashion. The plasmid pSL1142 (*Vch*INTEGRATE) Addgene #160730, a gift from Samuel H. Sternberg, was used as the backbone to assemble the components of the *Vpa*INTEGRATE system. We first PCR amplified the CRISPR array, the *tnsABC* loci, and the cargo from *Vch*INTEGRATE to generate three modular constructs (pDZ535, pDZ583, and pDZ551) in the cloning plasmid pJET 1.2 Blunt. These constructs have restriction sites that can be used for the final assembly into the backbone of pDZ387 (pSL1142).

We used site-directed mutagenesis with the Q5 site-directed mutagenesis kit from New England Biolabs to convert the *Vch*CAST typical CRISPR array in plasmid pDZ535 into the *Vpa*CAST typical CRISPR array generating plasmid pDZ633. To generate plasmid pDZ653, we cloned the *tniQcas876* genes from the *Vpa*CAST system in pDZ633 by means of an isothermal assembly reaction. The reaction consisted of the NEBuilder HiFi DNA Assembly Master Mix (New England Biolabs) and two PCR fragments, one corresponding to *tniQcas876* and the other corresponding to linearized pDZ633 obtained by inverted PCR. We added an ApaI restriction site downstream of *cas6* in pDZ653 to facilitate modular cloning of the *Vpa*CAST-*tniQcas876* region. Since the *Vpa*CAST *tniQ* gene has an internal BsaI restriction site that would interfere with the cloning of CRISPR spacers, we removed this restriction site by site-directed mutagenesis. We introduced a silent mutation in the codon for serine 231 in *Vpa tniQ,* changing it from tct to tca.

Plasmid pDZ744 was generated by exchanging the *Vch tnsABC* genes with the *Vpa tnsABC* genes into the linearized pDZ583 plasmid. We used a T4 polynucleotide kinase from New England Biolabs to phosphorylate the 5′ ends of the PCR products corresponding to *Vpa tnsABC* and linearized pDZ853. The phosphorylated PCR products were ligated using a T4 ligase from New England Biolabs. The *Vpa tnsABC* fragment in pDZ744 has NdeI and ApaI restriction sites upstream of *tnsA* that allow the cloning of the *Vpa tniQcas876* module through the ligation of sticky ends.

Plasmid pDZ661 was generated by replacing the *Vch*CAST R and L-terminal sequences from plasmid pDZ551 with the *Vpa*CAST R and L-terminal sequences, sequentially, using a similar blunt-cloning approach as the one described for the generation of pDZ744.

To assemble plasmid pDZ879 containing the *Vpa*INTEGRATE system, we first cloned the *Vpa* mini-CAST cargo into pDZ387 using Bsu36I and KpnI restriction sites to generate plasmid pDZ681. We next cloned the *Vpa tnsABC* module using NdeI and Bsu36I restriction sites within pDZ681 to generate pDZ799. Finally, pDZ799 was digested with the restriction enzymes SalI and ApaI to ligate the *Vpa* CRISPR-*tniQcas* fragment.

The allelic exchange of *lacZ_Vpa_* for *lacZ_Ec_* was done through double homologous recombination. First, the *lacZ_Ec_* allele was PCR amplified using genomic DNA of BL21 DE3 and cloned into a linearized pMMB67EH-Gen plasmid (pDZ510). This construct was used as a template to amplify the P_tac_-*lacZ_Ec_* region that was used for allele replacement. The suicide plasmid pDZ260 was used for allele replacement. This plasmid has 500 bp sequences that correspond to upstream and downstream homology regions flanking the *lacZ_Vpa_* allele. In between these homology regions is an FRT-*kanR*-FRT cassette followed by unique SacI and SpeI restriction sites. The Ptac-*lacZ_Ec_* region was cloned by isothermal assembly into pDZ260 linearized with SacI and SpeI to generate pDZ518. Allele exchange was achieved following the same protocol previously reported for the generation of gene deletions by double homologous recombination in *V. parahaemolyticus* (52, 56). The kanamycin resistance cassette was removed by site-specific recombination using plasmid pDZ514 that expresses the Flp recombinase and the counter selection allele *sacB*. To make pDZ514 and pDZ527, the *flp* cassette was amplified from plasmid pCP20 (57) and the *sacB* cassette from pRE118. The *flp* cassette consists of the *flp* gene and a gene that encodes a temperature-sensitive λ repressor gene. Both genes are controlled by a single λ promoter. The *flp* cassette and the *sacB* cassette were assembled into either a pBBR1MCS-5 (pDZ514) or pBBR1MCS-2 (pDZ527) backbone through isothermal assembly. Site-specific recombination was induced by incubating exponentially grown cells at 42°C for 1 to 2 hours. The desired eliminations were validated by PCR.

The deletion of the *Vpa*CAST system from chromosome 2 in *V. parahaemolyticus* RIMD2210633 was achieved by a combination of homologous and site-specific recombination approaches. The L-terminal sequence was eliminated by double homologous recombination using plasmid pDZ759. To generate pDZ759 regions of approximately 500 bp flanking the L-terminal sequence from VpaI-7, they were cloned into the suicide plasmid pRE118. Deletion of the L-terminal sequence from VpaI-7 by double homologous recombination was achieved as previously reported (52, 56). To delete a region from VPA1387 (*tniQ*) to VPA1397 (*yciA*), an FRT-*genR* gentamicin resistance cassette was inserted upstream of *tniQ,* and an FRT-*sfgfp* cassette was inserted within VPA1397. This was achieved by double homologous recombination following a similar approach as the one used to delete the L-terminal sequence. Flp site-specific recombination was used to delete the region flanked by the FRT-*genR* and the FRT-*sfgfp* cassettes.

### Luminescence assay

The luminescence assays in planktonic cultures were performed as previously described (52, 56). To quantify light production in planktonic cells, we diluted cultures grown overnight 1:200 in 5 mL of LB broth in test tubes with a volumetric capacity of 20 mL and incubated them under shaking conditions (200 rpm) at 30°C until reaching an optical density at 600 nm (OD600) between 0.6 and 0.8. Light production from cells transferred to white 96-well plates was detected using a Synergy H1 plate reader from BioTek. The relative luminescent units (RLU) are calculated as arbitrary luminescence per mL and divided by the OD_600_ of the cultures.

### Generation of a consensus logo for the putative binding site of VpxR

To find conserved motifs, we used 300 nucleotides of the upstream sequence of *vpxR* and orthologs from *Aliivibrio salmonicida* LFI1238*, Vibrio vulnificus* CMCP6, *Vibrio campbellii* ATCC BAA-1116, and *Vibrio cholerae* O395 and 84 nucleotides of the upstream sequence of the *Vpa*CAST atypical CRISPR array and homologous atypical CRISPR arrays identified in the genomes of *Vibrio diabolicus* FDAARGOS 105 and *Vibrio natriegens* CCUG 16374. Motifs and their consensus logo were generated using the Multiple Em for Motif Elicitation (MEME) algorithm (58).

### Generation of reprogrammed CRISPR arrays in the INTEGRATE systems

Plasmids pDZ387 and pDZ879 have a non-targeting spacer sequence with two internal BsaI restriction sites that can be used to clone targeting spacer sequences. The targeting spacer sequences were designed with a python script generated by the Sternberg Lab and available through Github (https://github.com/sternberglab/CAST-guide-RNA-tool). The targeting spacers are 32 bp long and were cloned as oligoduplexes in the backbone of pDZ387 or pDZ879. The oligoduplexes were generated by annealing complementary oligonucleotides. The annealing procedure was done in a 100 µL solution with each oligonucleotide at a 20 µM concentration in a buffer composed of 60 mM KCl, 6 mM HEPES-pH 7.5, and 0.2 mM MgCl2. The solution was incubated at 95°C for 1 minute and then cooled down to 25°C by reducing the temperature 1°C every minute in a Thermocycler. The annealed oligoduplexes were phosphorylated using a T4 polynucleotide kinase from New England Biolabs. The plasmids pDZ387 and pDZ879 were digested with BsaI-HFv2 from New England Biolabs. The phosphorylated oligoduplexes were ligated to the linearized pDZ387 and pDZ879 plasmids using a T4 DNA ligase.

### INTEGRATE-mediated transposition assays

To use a conjugation approach to mobilize the pDZ879, pSL1142, pDZ952, and pDZ1000 plasmids to both *E. coli* and *V. parahaemolyticus* strains, we generated an *E. coli* BL21 DE3 receiver strain with a selection marker. To do so, we introduced into *E. coli* BL21 DE3 the plasmid pBAMD1-6 (55), which has a mini-Tn5 transposon with a gentamicin resistance cassette. We selected a gentamicin-resistant colony (EcDZ1067) with a random transposition event that grew like the parental strain. The pSL1142-derivative plasmids were horizontally transferred to the EcDZ1067 and VpDZ838 strains by a triparental mating approach using the helper plasmid pRK600 as previously reported (56). EcDZ1067-derivative transconjugants were selected in LB agar plates with gentamicin and kanamycin, while VpDZ838-derivative transconjugants were selected in LB agar plates with streptomycin and kanamycin. *E. coli* and *V. parahaemolyticus* strains were incubated at 30°C for 48 and 24 hours, respectively. X-gal at 40 µg/mL and IPTG at 0.1 mM were added to LB agar plates when indicated. The blue vs white colony count was done from an average of 150 colonies per plate.

Insertion mutations using the *Vch*INTEGRATE system from plasmid pSL1142 (pDZ387) were generated similarly as described above. The pDZ387-derivative plasmids with a reprogrammed targeting spacer were mobilized into *V. parahaemolyticus* RIMD2210633 through triparental mating. Transconjugants were selected in LB agar plates with streptomycin and kanamycin and incubated for 48 hours at 30°C. The mini-CAST insertion was analyzed with specific primers. The pDZ387-derivative plasmids were curated by growing the insertion mutants without selection twice. Kanamycin-sensitive colonies were genotyped by colony PCR using a MyTaq polymerase Mix from Meridian Bioscience.

## References

[1] Gavilan RG, Caro-Castro J, Blondel CJ, Martinez-Urtaza J. 2023. Vibrio parahaemolyticus epidemiology and pathogenesis: novel insights on an emerging foodborne pathogen. Adv Exp Med Biol 1404:233–251. doi:10.1007/978-3-031-22997-8_12 36792879

[2] Boyd EF, Cohen ALV, Naughton LM, Ussery DW, Binnewies TT, Stine OC, Parent MA. 2008. Molecular analysis of the emergence of pandemic Vibrio parahaemolyticus. BMC Microbiol 8:110. doi:10.1186/1471-2180-8-110 18590559 PMC2491623

[3] Li L, Meng H, Gu D, Li Y, Jia M. 2019. Molecular mechanisms of Vibrio parahaemolyticus pathogenesis. Microbiol Res 222:43–51. doi:10.1016/j.micres.2019.03.003 30928029

[4] Ceccarelli D, Hasan NA, Huq A, Colwell RR. 2013. Distribution and dynamics of epidemic and pandemic Vibrio parahaemolyticus virulence factors. Front Cell Infect Microbiol 3:97. doi:10.3389/fcimb.2013.00097 24377090 PMC3858888

[5] Hurley CC, Quirke AM, Reen FJ, Boyd EF. 2006. Four genomic islands that mark post-1995 pandemic Vibrio parahaemolyticus isolates. BMC Genomics 7:1–19. doi:10.1186/1471-2164-7-104 16672049 PMC1464126

[6] Espejo RT, García K, Plaza N. 2017. Insight into the origin and evolution of the Vibrio parahaemolyticus pandemic strain. Front Microbiol 8:1397. doi:10.3389/fmicb.2017.01397 28790994 PMC5523582

[7] Jerez SA, Plaza N, Bravo V, Urrutia IM, Blondel CJ. 2023. Vibrio type III secretion system 2 is not restricted to the Vibrionaceae and encodes differentially distributed repertoires of effector proteins. Microb Genom 9:1–15. doi:10.1099/mgen.0.000973 PMC10210961 37018030

[8] Okada N, Matsuda S, Matsuyama J, Park K-S, de los Reyes C, Kogure K, Honda T, Iida T. 2010. Presence of genes for type III secretion system 2 in Vibrio mimicus strains. BMC Microbiol 10:1–10. doi:10.1186/1471-2180-10-302 21110901 PMC3004890

[9] Peters JE, Makarova KS, Shmakov S, Koonin EV. 2017. Recruitment of CRISPR-Cas systems by Tn7-like transposons. Proc Natl Acad Sci U S A 114:E7358–E7366. doi:10.1073/pnas.1709035114 28811374 PMC5584455

[10] Tenjo-Castaño F, Montoya G, Carabias A. 2023. Transposons and CRISPR: rewiring gene editing. Biochemistry 62:3521–3532. doi:10.1021/acs.biochem.2c00379 36130724 PMC10734217

[11] Koonin EV, Krupovic M. 2023. New faces of prokaryotic mobile genetic elements: guide RNAs link transposition with host defense mechanisms. Curr Opin Syst Biol 36:100473. doi:10.1016/j.coisb.2023.100473 37779558 PMC10538440

[12] Hsieh SC, Peters JE. 2024. Natural and engineered guide RNA-directed transposition with CRISPR-associated Tn7-like transposons. Annu Rev Biochem 93:139–161. doi:10.1146/annurev-biochem-030122-041908 38598855 PMC11406308

[13] McDonald ND, Regmi A, Morreale DP, Borowski JD, Boyd EF. 2019. CRISPR-Cas systems are present predominantly on mobile genetic elements in Vibrio species. BMC Genomics 20:105. doi:10.1186/s12864-019-5439-1 30717668 PMC6360697

[14] Zhang E, Zhou W, Zhou J, He Z, Zhou Y, Han J, Qu D. 2022. CRISPR-Cas systems are present predominantly on chromosome and its relationship with MEGs in Vibrio species. Arch Microbiol 204:1–12. doi:10.1007/s00203-021-02656-1 34953139

[15] Hu K, Chou CW, Wilke CO, Finkelstein IJ. 2024. Distinct horizontal transfer mechanisms for type I and type V CRISPR-associated transposons. Nat Commun 15:6653. doi:10.1038/s41467-024-50816-w 39103341 PMC11300857

[16] Klompe SE, Vo PLH, Halpin-Healy TS, Sternberg SH. 2019. Transposon-encoded CRISPR-Cas systems direct RNA-guided DNA integration. Nature 571:219–225. doi:10.1038/s41586-019-1323-z 31189177

[17] Peters JE, Fricker AD, Kapili BJ, Petassi MT. 2014. Heteromeric transposase elements: generators of genomic islands across diverse bacteria. Mol Microbiol 93:1084–1092. doi:10.1111/mmi.12740 25091064

[18] Choi KY, Li Y, Sarnovsky R, Craig NL. 2013. Direct interaction between the TnsA and TnsB subunits controls the heteromeric Tn7 transposase. Proc Natl Acad Sci U S A 110:E2038–E2045. doi:10.1073/pnas.1305716110 23674682 PMC3670325

[19] Choi KY, Spencer JM, Craig NL. 2014. The Tn7 transposition regulator TnsC interacts with the transposase subunit TnsB and target selector TnsD. Proc Natl Acad Sci U S A 111:E2858–E2865. doi:10.1073/pnas.1409869111 24982178 PMC4104917

[20] Shen Y, Gomez-Blanco J, Petassi MT, Peters JE, Ortega J, Guarné A. 2022. Structural basis for DNA targeting by the Tn7 transposon. Nat Struct Mol Biol 29:143–151. doi:10.1038/s41594-022-00724-8 35173349 PMC12747246

[21] Shen Y, Krishnan SS, Petassi MT, Hancock MA, Peters JE, Guarné A. 2024. Assembly of the Tn7 targeting complex by a regulated stepwise process. Mol Cell 84:2368–2381. doi:10.1016/j.molcel.2024.05.012 38834067 PMC11364213

[22] Petassi MT, Hsieh SC, Peters JE. 2020. Guide RNA categorization enables target site choice in Tn7-CRISPR-Cas transposons. Cell 183:1757–1771. doi:10.1016/j.cell.2020.11.005 33271061 PMC7770071

[23] Klompe SE, Jaber N, Beh LY, Mohabir JT, Bernheim A, Sternberg SH. 2022. Evolutionary and mechanistic diversity of Type I-F CRISPR-associated transposons. Mol Cell 82:616–628. doi:10.1016/j.molcel.2021.12.021 35051352 PMC8849592

[24] Baliga P, Shekar M, Ahamed ST, Venugopal MN. 2019. Antibiotic resistance pattern and its correlation to the presence of TDH gene and CRISPR-Cas system in Vibrio parahaemolyticus strains isolated from seafood. Indian J Fish 66:108. doi:10.21077/ijf.2019.66.2.86920-14

[25] Jingjit N, Preeprem S, Surachat K, Mittraparp-Arthorn P. 2021. Characterization and analysis of clustered regularly interspaced short palindromic repeats (CRISPRs) in pandemic and non-pandemic Vibrio parahaemolyticus isolates from seafood sources. Microorganisms 9:1220. doi:10.3390/microorganisms9061220 34199972 PMC8226915

[26] Vo PLH, Ronda C, Klompe SE, Chen EE, Acree C, Wang HH, Sternberg SH. 2021. CRISPR RNA-guided integrases for high-efficiency, multiplexed bacterial genome engineering. Nat Biotechnol 39:480–489. doi:10.1038/s41587-020-00745-y 33230293 PMC10583764

[27] Solovyev V, Salamov A. 2011. Automatic annotation of microbial genomes and metagenomic sequences, p 62–78. In Metagenomics and its applications in agriculture, biomedicine and environmental studies.

[28] Feklístov A, Sharon BD, Darst SA, Gross CA. 2014. Bacterial sigma factors: a historical, structural, and genomic perspective. Annu Rev Microbiol 68:357–376. doi:10.1146/annurev-micro-092412-155737 25002089

[29] Goss TJ, Morgan SJ, French EL, Krukonis ES. 2013. ToxR recognizes a direct repeat element in the toxT, ompU, ompT, and ctxA promoters of Vibrio cholerae to regulate transcription. Infect Immun 81:884–895. doi:10.1128/IAI.00889-12 23297386 PMC3584884

[30] Bina XR, Howard MF, Ante VM, Bina JE. 2016. Vibrio cholerae LeuO links the ToxR regulon to expression of lipid a remodeling genes. Infect Immun 84:3161–3171. doi:10.1128/IAI.00445-16 27550934 PMC5067749

[31] Ante VM, Bina XR, Howard MF, Sayeed S, Taylor DL, Bina JE. 2015. Vibrio cholerae leuO transcription is positively regulated by toxr and contributes to bile resistance. J Bacteriol 197:3499–3510. doi:10.1128/JB.00419-15 26303831 PMC4621094

[32] Whitaker WB, Parent MA, Boyd A, Richards GP, Boyd EF. 2012. The Vibrio parahaemolyticus ToxRS regulator is required for stress tolerance and colonization in a novel orogastric streptomycin-induced adult murine model. Infect Immun 80:1834–1845. doi:10.1128/IAI.06284-11 22392925 PMC3347455

[33] Gode-Potratz CJ, Chodur DM, McCarter LL. 2010. Calcium and iron regulate swarming and type III secretion in Vibrio parahaemolyticus. J Bacteriol 192:6025–6038. doi:10.1128/JB.00654-10 20851895 PMC2976450

[34] Kazi MI, Conrado AR, Mey AR, Payne SM, Davies BW. 2016. ToxR antagonizes H-NS regulation of horizontally acquired genes to drive host colonization. PLoS Pathog 12:e1005570. doi:10.1371/journal.ppat.1005570 27070545 PMC4829181

[35] Hernández-Lucas I, Gallego-Hernández AL, Encarnación S, Fernández-Mora M, Martínez-Batallar AG, Salgado H, Oropeza R, Calva E. 2008. The LysR-type transcriptional regulator LeuO controls expression of several genes in Salmonella enterica serovar Typhi. J Bacteriol 190:1658–1670. doi:10.1128/JB.01649-07 18156266 PMC2258680

[36] Shimada T, Bridier A, Briandet R, Ishihama A. 2011. Novel roles of LeuO in transcription regulation of E.coli genome: antagonistic interplay with the universal silencer H-NS. Mol Microbiol 82:378–397. doi:10.1111/j.1365-2958.2011.07818.x 21883529

[37] Boas Lichty KE, Gregory GJ, Boyd EF. 2023. NhaR, LeuO, and H-NS are part of an expanded regulatory network for ectoine biosynthesis expression. Appl Environ Microbiol 89:e0047923. doi:10.1128/aem.00479-23 37278653 PMC10304999

[38] Madeira F, Madhusoodanan N, Lee J, Eusebi A, Niewielska A, Tivey ARN, Lopez R, Butcher S. 2024. The EMBL-EBI Job Dispatcher sequence analysis tools framework in 2024. Nucleic Acids Res 52:W521–W525. doi:10.1093/nar/gkae241 38597606 PMC11223882

[39] Hoffmann FT, Kim M, Beh LY, Wang J, Vo PLH, Gelsinger DR, George JT, Acree C, Mohabir JT, Fernández IS, Sternberg SH. 2022. Selective TnsC recruitment enhances the fidelity of RNA-guided transposition. Nature 609:384–393. doi:10.1038/s41586-022-05059-4 36002573 PMC10583602

[40] Hsieh SC, Peters JE. 2023. Discovery and characterization of novel type I-D CRISPR-guided transposons identified among diverse Tn7-like elements in cyanobacteria. Nucleic Acids Res 51:765–782. doi:10.1093/nar/gkac1216 36537206 PMC9881144

[41] Saito M, Ladha A, Strecker J, Faure G, Neumann E, Altae-Tran H, Macrae RK, Zhang F. 2021. Dual modes of CRISPR-associated transposon homing. Cell 184:2441–2453. doi:10.1016/j.cell.2021.03.006 33770501 PMC8276595

[42] Strecker J, Ladha A, Gardner Z, Schmid-Burgk JL, Makarova KS, Koonin EV, Zhang F. 2019. RNA-guided DNA insertion with CRISPR-associated transposases. Science 365:48–53. doi:10.1126/science.aax9181 31171706 PMC6659118

[43] Wang S, Gabel C, Siddique R, Klose T, Chang L. 2023. Molecular mechanism for Tn7-like transposon recruitment by a type I-B CRISPR effector. Cell 186:4204–4215. doi:10.1016/j.cell.2023.07.010 37557170 PMC11027886

[44] Walker MWG, Klompe SE, Zhang DJ, Sternberg SH. 2023. Novel molecular requirements for CRISPR RNA-guided transposition. Nucleic Acids Res 51:4519–4535. doi:10.1093/nar/gkad270 37078593 PMC10201428

[45] Lewis D, Le P, Zurla C, Finzi L, Adhya S. 2011. Multilevel autoregulation of λ repressor protein CI by DNA looping in vitro. Proc Natl Acad Sci U S A 108:14807–14812. doi:10.1073/pnas.1111221108 21873207 PMC3169136

[46] Ptashne M, Backman K, Humayun MZ, Jeffrey A, Maurer R, Meyer B, Sauer RT. 1976. Autoregulation and function of a repressor in bacteriophage lambda. Science 194:156–161. doi:10.1126/science.959843 959843

[47] Meyer BJ, Kleid DG, Ptashne M. 1975. Lambda repressor turns off transcription of its own gene. Proc Natl Acad Sci U S A 72:4785–4789. doi:10.1073/pnas.72.12.4785 1061069 PMC388816

[48] Sharpe PL, Craig NL. 1998. Host proteins can stimulate Tn7 transposition: a novel role for the ribosomal protein L29 and the acyl carrier protein. EMBO J 17:5822–5831. doi:10.1093/emboj/17.19.5822 9755182 PMC1170910

[49] Lampe GD, King RT, Halpin-Healy TS, Klompe SE, Hogan MI, Vo PLH, Tang S, Chavez A, Sternberg SH. 2024. Targeted DNA integration in human cells without double-strand breaks using CRISPR-associated transposases. Nat Biotechnol 42:87–98. doi:10.1038/s41587-023-01748-1 36991112 PMC10620015

[50] Schmitz M, Querques I, Oberli S, Chanez C, Jinek M. 2022. Structural basis for the assembly of the type V CRISPR-associated transposon complex. Cell 185:4999–5010. doi:10.1016/j.cell.2022.11.009 36435179 PMC9798831

[51] Tenjo-Castaño F, Sofos N, Stutzke LS, Temperini P, Fuglsang A, Pape T, Mesa P, Montoya G. 2024. Conformational landscape of the type V-K CRISPR-associated transposon integration assembly. Mol Cell 84:2353–2367. doi:10.1016/j.molcel.2024.05.005 38834066

[52] Martínez-Méndez R, Camacho-Hernández DA, Sulvarán-Guel E, Zamorano-Sánchez D. 2021. A trigger phosphodiesterase modulates the global c-di-GMP pool, motility, and biofilm formation in Vibrio parahaemolyticus. J Bacteriol 203:e0004621. doi:10.1128/JB.00046-21 33846117 PMC8316121

[53] Edwards RA, Keller LH, Schifferli DM. 1998. Improved allelic exchange vectors and their use to analyze 987P fimbria gene expression. Gene 207:149–157. doi:10.1016/s0378-1119(97)00619-7 9511756

[54] Lenz DH, Mok KC, Lilley BN, Kulkarni RV, Wingreen NS, Bassler BL. 2004. The small RNA chaperone Hfq and multiple small RNAs control quorum sensing in Vibrio harveyi and Vibrio cholerae. Cell 118:69–82. doi:10.1016/j.cell.2004.06.009 15242645

[55] Martínez-García E, Aparicio T, de Lorenzo V, Nikel PI. 2014. New transposon tools tailored for metabolic engineering of gram-negative microbial cell factories. Front Bioeng Biotechnol 2:46. doi:10.3389/fbioe.2014.00046 25389526 PMC4211546

[56] Zamorano-Sánchez D, Alejandre-Sixtos JE, Arredondo-Hernández A, Martínez-Méndez R. 2023. OpaR exerts a dynamic control over c-di-GMP homeostasis and cpsA expression in Vibrio parahaemolyticus through its regulation of ScrC and the trigger phosphodiesterase TpdA. Microbiol Spectr 11:e0087223. doi:10.1128/spectrum.00872-23 37199626 PMC10269520

[57] Datsenko KA, Wanner BL. 2000. One-step inactivation of chromosomal genes in Escherichia coli K-12 using PCR products. Proc Natl Acad Sci U S A 97:6640–6645. doi:10.1073/pnas.120163297 10829079 PMC18686

[58] Bailey TL, Elkan C. 1994. Fitting a mixture model by expectation maximization to discover motifs in biopolymers. Proceedings of the 2nd International Conference on Intelligent Systems for Molecular Biology, ISMB. p 28–36 7584402

